# Spiropyran-based chromic hydrogels for CO_2_ absorption and detection

**DOI:** 10.3389/fchem.2023.1176661

**Published:** 2023-05-23

**Authors:** Arnau Marco, Gonzalo Guirado, Rosa María Sebastián, Jordi Hernando

**Affiliations:** Departament de Química, Universitat Autònoma de Barcelona, Cerdanyola del Vallès, Spain

**Keywords:** spiropyran, hydrogel, CO_2_ detection, photochromism, acidochromism

## Abstract

By enabling rapid, cost-effective, user-friendly and *in situ* detection of carbon dioxide, colorimetric CO_2_ sensors are of relevance for a variety of fields. However, it still remains a challenge the development of optical chemosensors for CO_2_ that combine high sensitivity, selectivity and reusability with facile integration into solid materials. Herein we pursued this goal by preparing hydrogels functionalized with spiropyrans, a well-known class of molecular switches that undergo different color changes upon application of light and acid stimuli. By varying the nature of the substituents of the spiropyran core, different acidochromic responses are obtained in aqueous media that allow discriminating CO_2_ from other acid gases (e.g., HCl). Interestingly, this behavior can be transferred to functional solid materials by synthesizing polymerizable spiropyran derivatives, which are used to prepare hydrogels. These materials preserve the acidochromic properties of the incorporated spiropyrans, thus leading to selective, reversible and quantifiable color changes upon exposure to different CO_2_ amounts. In addition, CO_2_ desorption and, therefore, recovery of the initial state of the chemosensor is favored by irradiation with visible light. This makes spiropyran-based chromic hydrogels promising systems for the colorimetric monitorization of carbon dioxide in a diversity of applications.

## 1 Introduction

Spiropyrans (SPs) are amongst the most preferred molecular switches for the preparation of smart materials (Berkovic et al., 2000; Klajn, 2014; Kortekaas and Browne, 2019; Rad et al., 2022). This is mainly due to their extremely versatile switching behavior, as SPs are sensitive to a large variety of external stimuli. On the one hand, they are well known to reversibly photoconvert from their colorless ring-open spiranic form (Sp) to their colored ring-closed merocyanine isomer (Mc) via C-O bond light-induced cleavage and C=C isomerization, a behavior that can also be promoted thermally, mechanically and by varying solvent polarity (Kortekaas and Browne, 2019). In addition, SPs also respond to protonation, metal complexation and redox-induced electrodimerization processes (Kumar et al., 2021; Santiago et al., 2021). As a result, they can be interconverted between different states with clearly distinguishable physico-chemical properties, a feature that can be exploited in a diversity of areas ranging from information storage and processing (Berkovic et al., 2000; Andréasson and Pischel, 2015; Xia et al., 2022) to (bio)imaging (Zhu et al., 2006; Zhu et al., 2014; Qi et al., 2017), polymer actuators (Florea et al., 2012; Li et al., 2020; Pandurangan et al., 2021), drug delivery (Jia et al., 2018; Fagan et al., 2021) and photocontrollable biological systems (Sakata et al., 2005; Andersson et al., 2008; Bautista-Barrufet et al., 2014). Another relevant area of application of SPs is in (bio)sensing, where their multiresponsive chromic and fluorochromic properties can be utilized to optically report on mechanical damage in polymer materials (Li et al., 2018; Chen et al., 2021), temperature (Pietsch et al., 2011; Julià-López et al., 2016), UV exposure (Leinen et al., 2020) and a plethora of chemical analytes (e.g., metal ions, vapors, pH) (Quin et al., 2015; Prakash et al., 2016; Sahoo et al., 2018; Ali et al., 2020; Mandal et al., 2022). The latter is the interest of this work, where we present the development of novel SP-based materials for colorimetric CO_2_ sensing.

Optical CO_2_ detection has been proposed as an alternative to current commercial CO_2_ sensors based on IR spectroscopy, potentiometry and GC-MS, which might be instrumentally complex, expensive and nonselective (Guo et al., 2012). By contrast, colorimetric and fluorometric methods for CO_2_ monitorization can provide smaller sizes, potentially low prices and the capability for *in situ* analysis in a wide spectrum of applications (Guo et al., 2012; Xu et al., 2013; Xia et al., 2015; Lu et al., 2016)—e.g., greenhouse gas emission control (Shamsuzzaman et al., 2021), food preservation (Puligundla et al., 2012), beverage fermentation (Shen et al., 2004) and work health and safety (Allen et al., 2016). However, it still remains a challenge the development of optical chemosensors for CO_2_ that combine high sensitivity and selectivity with facile integration into solid materials (Chatterjee and Sen, 2015; Guo et al., 2019). Herein we envisage to reach this objective by means of hydrogels functionalized with SP dyes.

To date the use of SPs for optical CO_2_ detection has been scarce and mainly circumscribed to liquid solutions and suspensions. On the one hand, the well-known interaction between carbon dioxide and amidines in protic solvents has been used to induce color changes in SPs upon CO_2_ exposure in solution, either by incorporating amidine groups to their structure (Darwish et al., 2011) or as external additives (Darwish et al., 2010; Boyd et al., 2013; Shin et al., 2022). In a different approach, a mechanochromic SP was anchored in polyacrylate nanoparticles with pending amino groups, which swelled and changed their color when treated with CO_2_ in liquid suspension (Li et al., 2016). However, no attempts to transfer these strategies to solid materials have been described, which is an essential requirement to eventually accomplish sensing device fabrication. To our knowledge, only two precedents of the incorporation of SPs in solid matrices for CO_2_ detection can be found in the literature. In both reports, nitrospiropyran derivatives were attached to organic polymer nanofibers, which underwent a yellow-to-red color variation when in contact with aqueous CO_2_ that was ascribed to SP protonation (Shang et al., 2017; Dekhordi et al., 2021). But even in these cases, immersion in water (or previous wetting for prolonged times) of the SP-functionalized polymers was needed for CO_2_ detection, as it requires carbonic acid formation. In addition, no proof was given about the sensing selectivity of these materials for CO_2_ relative to other gaseous or dissolved acids (e.g., HCl), which are also known to promote the acidochromic response of nitrospiropyrans within polymer matrices (Nam et al., 2014; Genovese et al., 2015; Genovese et al., 2017; Rad et al., 2020; Guo et al., 2021; Xie et al., 2022). In those cases, the formation of a yellow protonated SP species was instead observed upon acid exposure, which is in better agreement with the well-established behavior of nitrospiropyrans in solution (Kortekaas et al., 2018; Kortekaas and Browne, 2019).

**FIGURE 1:**
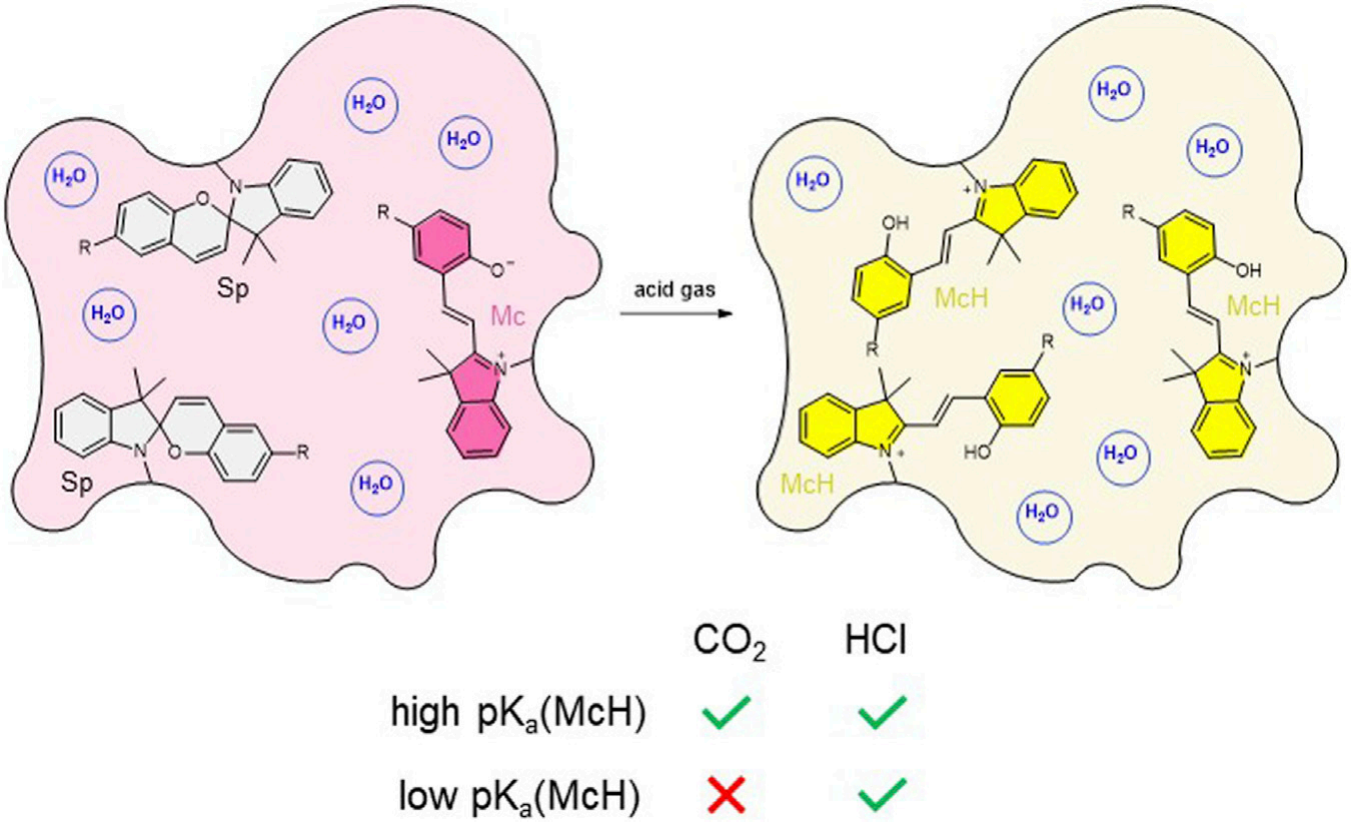
Strategy devised in this work for the sensing of gaseous CO_2_ with SP-based hydrogels. CO_2_-induced protonation of the initial Sp-Mc equilibrium mixture should only take place for hydrogels functionalized with SPs exhibiting high pK_a_ (McH). By contrast, other stronger and more water-soluble acid gases such as HCl should induce McH formation for all acidochromic SPs.

To address these issues on route to the development of self-standing solid materials that could directly sense gaseous CO_2_ selectively, herein we propose an alternative strategy that relies on two main design principles (Figure 1). First, acidochromic SPs will be incorporated into polymer hydrogels, which in contact with CO_2_ should intrinsically generate carbonic acid without the need of additional liquid media. As a result, the initial equilibrium mixture of their colorless Sp and colored Mc isomers should convert into the corresponding protonated Mc form (McH), which exhibits a different measurable absorption. In this way, colorimetric CO_2_ detection could take place directly on the gas-gel interface. Second, separate hydrogels will be prepared that contain SPs with different acid-base properties–i.e., distinct pK_a_ values for their protonated McH state (pK_a_ (McH)). Consequently, this should allow us discriminating the acidochromic signals measured for CO_2_ relative to other gases of differential acidity.

## 2 Results and discussion

### 2.1 Synthesis and acidochromic response of water-soluble spiropyrans

To accomplish selective colorimetric CO_2_ sensing with SP-based hydrogels, we aim to take advantage of the tunable acidochromic behavior of spiropyrans depending on their substituents (Kortekaas et al., 2018; Berton et al., 2020; Wimberger et al., 2021a; Berton et al., 2021; Wimberger et al., 2021b). As aqueous media saturated with CO_2_ present pH ∼ 4 at ambient conditions (Haghi et al., 2017), this means that large variation of SP protonation and, therefore, maximal color changes in our hydrogels when exposed to CO_2_ will only take place for spiropyran derivatives with pK_a_ (McH) ∼ 5–6. By contrast, if SP derivatives are used with pK_a_ (McH) < 4.5, minor or no color changes should be observed in the hydrogels upon CO_2_ absorption, whereas a distinct acidochromic response would be triggered by other acid gases that are more soluble (and stronger) in water–e.g., HCl, formic acid and acetic acid.

**SCHEME 1:**
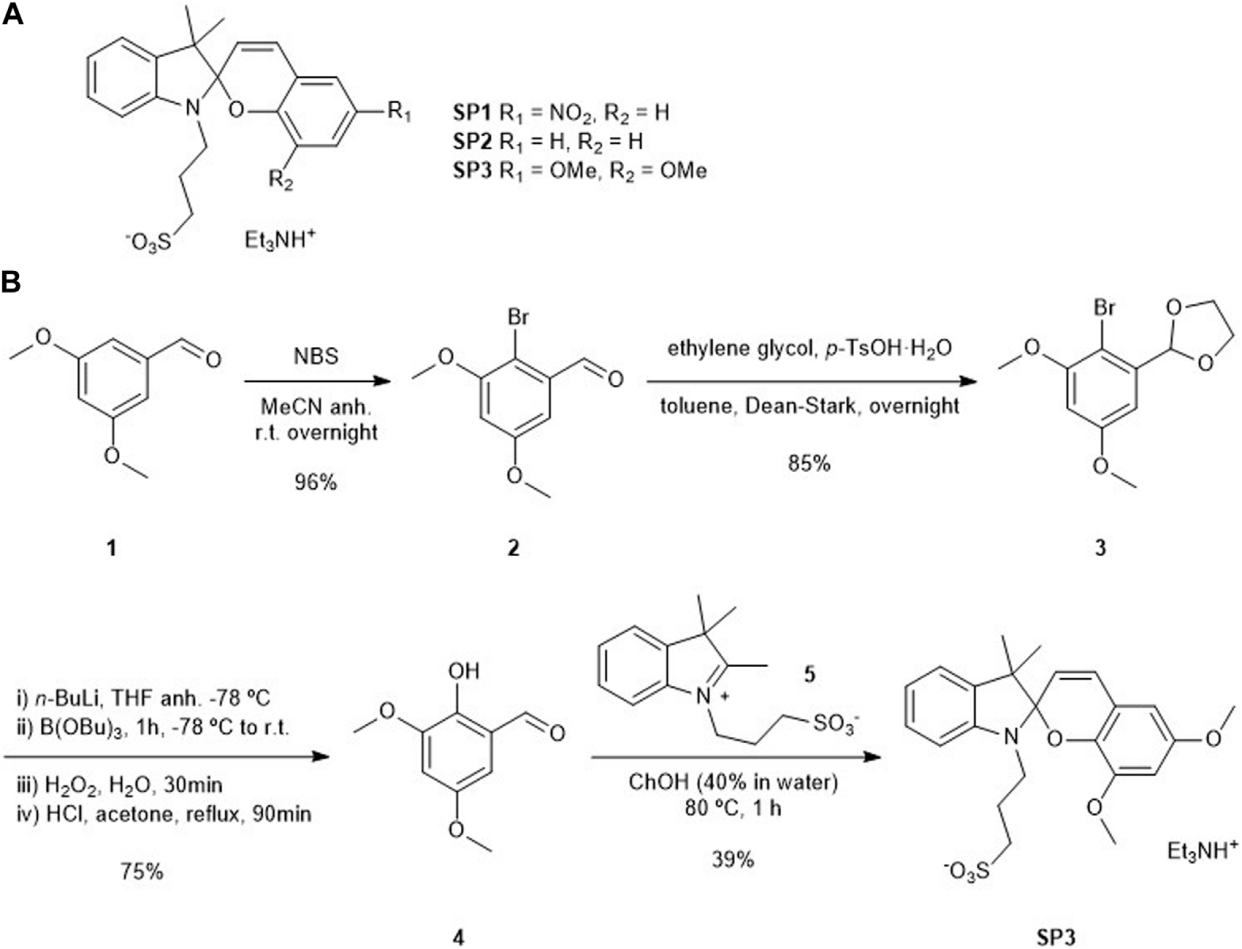
**(A)** Structures of water-soluble acidochromic SP1-SP3. **(B)** Synthesis of SP3.

To test this hypothesis, we decided to investigate the acidochomic response in aqueous solution of three different water-soluble SPs (SP1-SP3, Scheme 1A). SP1 and SP2 were chosen because of their clearly different pK_a_ (McH) values that should lead to distinct CO_2_-induced acidochromic responses: pK_a_ (McH) ∼ 4.7 and 6.3 in the dark for SP1 and SP2, respectively (Wimberger et al., 2021a). In addition, SP1 resembles the nitrospiropyran dyes previously anchored to polymer fibers for CO_2_ detection in aqueous media (Shang et al., 2017; Dekhordi et al., 2021). As for SP3, it is a novel SP derivative that was designed to further increase pK_a_ (McH) relative to SP2. For this purpose, two electro-donating methoxy groups were introduced to the 6 and 8 positions of the benzopyran moiety of the compound, which should further destabilize the negative charge of the phenolate moiety in the Mc isomer and, therefore, decrease the acidity of its protonated McH form.

For the synthesis of SP3, we followed the classical route for SP preparation, which required condensation of 2-hydroxybenzaldehyde **4** with indolium **5** (Scheme 1B, see Supplementary Figures S1–S9). While the latter was obtained as previously reported (Shi et al., 2011), a new synthetic route had to be devised to prepare **4**. It started with the 2-bromination of commercially available 3,5-dimetoxybenzaldehyde (**1**) with *N*-bromosuccinimide (NBS), which was followed by protection of the aldehyde group as a cyclic acetal. The resulting intermediate **3** was reacted with *n-*BuLi and B(OBu)_3_ to yield a boronic ester that could then be oxidized to the corresponding alcohol in the presence of hydrogen peroxide. Further acid treatment for aldehyde deprotection furnished the desired benzaldehyde **4**. Finally, condensation of this compound with indolium **5** mediated by choline hydroxide (ChOH) yielded the target spiropyran SP3 in a 24% overall yield.

**FIGURE 2:**
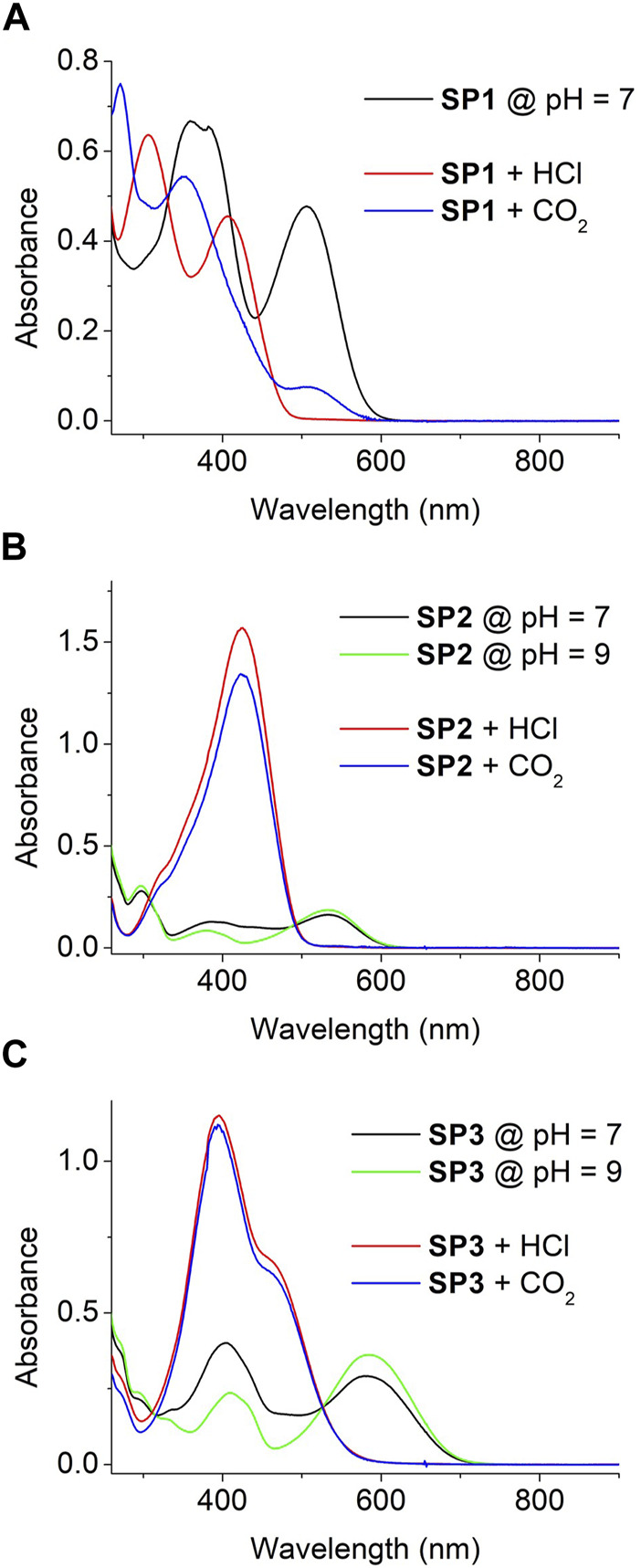
**(A)** UV-vis absorption spectra of SP1 (c = 1.7 10^−5^ M) in water at pH = 7 and after addition of HCl_**(g)**_ and CO_2**(g)**_ until saturation. **(B,C)** UV-vis absorption of SP2 (c = 4.7 10^−5^ M) and SP3 (c = 3.6 10^−5^ M) in water at pH = 7, pH = 9 and after addition of HCl_**(g)**_ and CO_2**(g)**_ until saturation. In all the cases, HCl_**(g)**_ or CO2_**(g)**_ flows were bubbled through the solutions until no more absorption changes were observed.

The UV-vis absorption spectra of SP1-SP3 were initially measured both in water at neutral pH (pH = 7, Figure 2) and in a polar nonaqueous solvent (MeOH, see Supplementary Figure S10). In both media, several different absorption bands were registered both in the UV and visible regions. As previously described for similar compounds (Kortekaas et al., 2018; Wimberger et al., 2021a), these bands can be mainly attributed to the coexistence of two different species in equilibrium: (a) their initial spiranic colorless Sp state, which shows maximum absorption at λ_max_ ∼ 300 (SP2) and 340 nm (SP1, SP3); and (b) their open Mc isomer exhibiting a longer conjugation path, which results in visible light absorption at λ_max_ ∼ 380 and 510 nm (SP1), 380 and 530 nm (SP2), and 410 and 590 nm (SP3). As a result, both the aqueous and MeOH solutions of SP1-SP3 presented pink-red (SP1, SP2) and blue (SP3) coloration. As expected, the intensity of their coloration at equal concentration–i.e., the absorbance ratio between Mc and Sp bands–increased in water at pH = 7 relative to MeOH. This can be ascribed to the larger stabilization of the charged phenolate moiety of Mc in aqueous media relative to organic solvents, which displaces the Sp-Mc equilibrium towards the formation of the colored ring-open isomer (Wimberger et al., 2021a).

The absorption spectra of SPs can be even further complicated by their photo- and acidochromic properties. Thus, irradiation of aqueous solutions of SP1 and SP2 with both UV and visible light in water resulted in reversible solution decoloration due to Mc → Sp photoconversion for both compounds (Wimberger et al., 2021a) (see Supplementary Figures S11A, B). Surprisingly, such effect was not observed for SP3 neither via steady-state nor time-resolved absorption measurements, which suggests that it is a nonphotoswitchable spiropyran (see Supplementary Figure S11C). A similar result was reported for other SP derivatives bearing strong mesomeric electron-donating groups (Balmond et al., 2016), which we tentatively attribute to the occurrence of photoinduced intramolecular charge transfer processes between the electron-poor indolium and electron-rich benzopyran rings upon irradiation that efficiently compete with photoisomerization. Of more interest for our work is the acidochromic behavior of SP1-SP3. In the case of SP1, no protonation effects should be expected in water at pH = 7 due to its low pK_a_ (McH) ∼ 4.7 (Wimberger et al., 2021a). By contrast, as pK_a_ (McH) ∼ 6.3 for SP2 (Wimberger et al., 2021a), a fraction of their molecules must be protonated to form the corresponding McH state in neutral aqueous media. This was easily proven by base addition until pH = 9, which resulted in a decrement of the absorption at ∼400–500 nm ascribed to McH (Figure 2B) - i.e., it led to full deprotonation of the system, which was converted into a pure equilibrium mixture of the Sp and Mc isomers of SP2. Interestingly, a similar result was observed for SP3 upon basification (Figure 2C). Clearly, this result suggests that the pK_a_ (McH) of SP3 lies closer to that of SP2 and, therefore, that it gives rise to a mixture of Sp, Mc and McH molecules upon dissolution in water at pH = 7.

**FIGURE 3:**
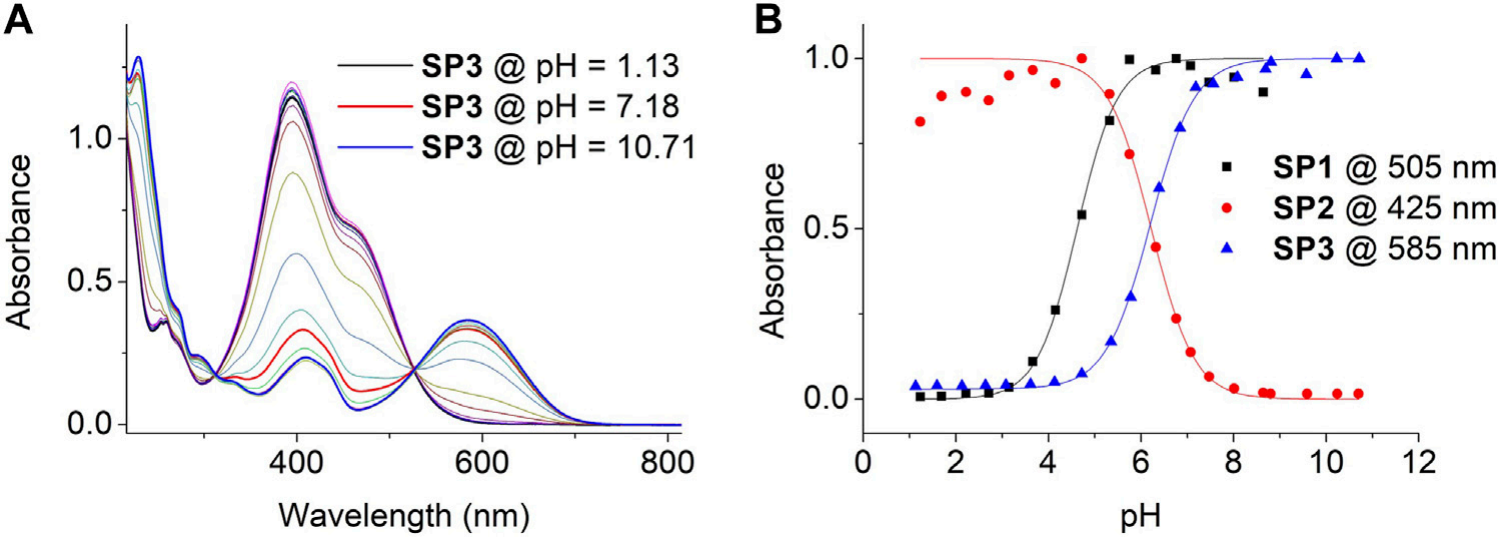
**(A)** Variation of the UV-vis absorption of SP3 in water with pH. Measurements were conducted by dissolving SP3 (c = 3.6 10^−5^ M) in independent buffer solutions of pH = 1.13, 1.59, 2.13, 2.64, 3.08, 3.61, 4.13, 4.72, 5.32, 5.78, 6.39, 6.84, 7.18, 7.56, 8.08, 8.69, 8.81, 9.57, 10.23 and 10.71. **(B)** pH-induced variation of the absorbance of SP1-SP3 in aqueous media at λ = 505 (SP1), 425 (SP2) and 585 nm (SP3). Symbols correspond to experimental data, while lines are fits to Eq. 1 for pK_a_ (McH) determination (see Section 4.4).

To get a deeper insight on the acidochromic behavior of SP1-SP3, the pH-induced variation of its UV-vis absorption spectra in water was carefully monitored in the dark (Figure 3A and Supplementary Figure S12). From this data, the pK_a_ (McH) of new compound SP3 was determined to be 6.17, while the values found for SP1 (pK_a_ (McH) = 4.63) and SP2 (pK_a_ (McH) = 6.23) reproduced those already described in the literature (Wimberger et al., 2021a) (Figure 3B and Supplementary Table S1). Although they characterize the acid-base behavior of SP1-SP3 in the dark in aqueous media, it must be noted that the experimentally determined pK_a_ (McH) values are not the intrinsic pK_a_ constants of the McH state of SP1-SP3 - i.e., the acidity constants of the pure McH-Mc ionization process (Berton et al., 2020; Wimberger et al., 2021a). This is due to the fact that, upon deprotonation of McH, its conjugate base Mc establishes a further thermal equilibrium with the spiranic Sp state that also affects the acid-base process (see Supplementary Scheme S1). By proper analysis of this equilibrium by ^1^H NMR or UV-vis spectroscopy, the intrinsic acidity constants of McH can also be determined (Berton et al., 2020; Wimberger et al., 2021a), which in our case were found to be pK_a,intrinsic_ (McH) = 4.82, 7.03 and 6.66 for SP1, SP2 and SP3, respectively (see Supplementary Table S1).

According to our results, the newly synthesized SP3 presents similar pK_a_ (McH) and pK_a,intrinsic_ (McH) values to SP2 and we could not make its McH form even less acidic. However, two important differences were observed for the acidochromic behavior of SP3 relative to SP2 that could be of interest for future works. First, when dissolved in aqueous media of variable pH, SP3 reaches its thermal Sp-Mc-McH equilibrium state very rapidly at room temperature (<1 min), while it takes much longer for SP2 (up to 15 min at pH ∼ 6). This indicates that the thermal ring-opening and ring-closing processes are significantly faster for SP3. Second, the acidochromic properties of SP3 are not affected by light irradiation, as the photoinduced isomerization of its McH form is also inhibited. This is in contrast with SP1 and SP2, whose McH state photoisomerizes upon irradiation to yield a more acidic *cis*-McH form (see Supplementary Figures S11D, F and Supplementary Scheme S1).

Because of the differences in pK_a_ (McH) between SP1 and SP2-SP3, their aqueous solutions should differently respond to acid gases of distinct solubility and strength in water. On the one hand, when HCl_(g)_ was bubbled to water solutions of SP1-SP3, similar changes were observed in all the cases: the typical Mc absorption band at *λ* > 500 nm fully disappeared, while intense absorption bands raised with λ_max_ ∼ 430 nm (Figure 2). As a result, all the solutions turned from pink-red (SP1-SP2) or blue (SP3) to yellow (SP1-SP2) or orange (SP3), which is in agreement with the complete protonation of SP molecules to yield McH - i.e., high acid media were produced by HCl dissolution whose pH must be even significantly lower than the pK_a_ (McH) of SP1. On the other hand, different acidochromic responses were obtained when the aqueous solutions of SP1-SP3 were saturated with CO_2(g)_. For SP2 and SP3 with larger pK_a_ (McH), absorption changes similar to those observed with HCl_(g)_ were registered - i.e., their nearly full protonation to produce the corresponding yellow- (SP2) and orange-colored (SP3) McH molecules. However, this was not the case for SP1 having a more acidic McH state. For this compound, the McH absorption spectrum was not retrieved after CO_2_ exposure, which indicates that insufficiently acid pH values were reached as to promote large SP protonation. Instead, other CO_2_-induced colorimetric changes were observed for SP1 in water: a variation of the relative intensity of the Sp and Mc absorption bands, which suggests a change in the composition of the Sp-Mc mixture after CO_2_ dissolution and can be related with the previous reports on CO_2_ detection using polymer fibers functionalized with similar nitrospiropyrans (Shang et al., 2017; Dekhordi et al., 2021). Overall, these results validate our strategy of exploiting SPs of contrasting pK_a_ (McH) values to discriminate the acidochromic responses caused by different gases.

### 2.2 Synthesis and acidochromic response of spiropyran-based hydrogels

**SCHEME 2:**
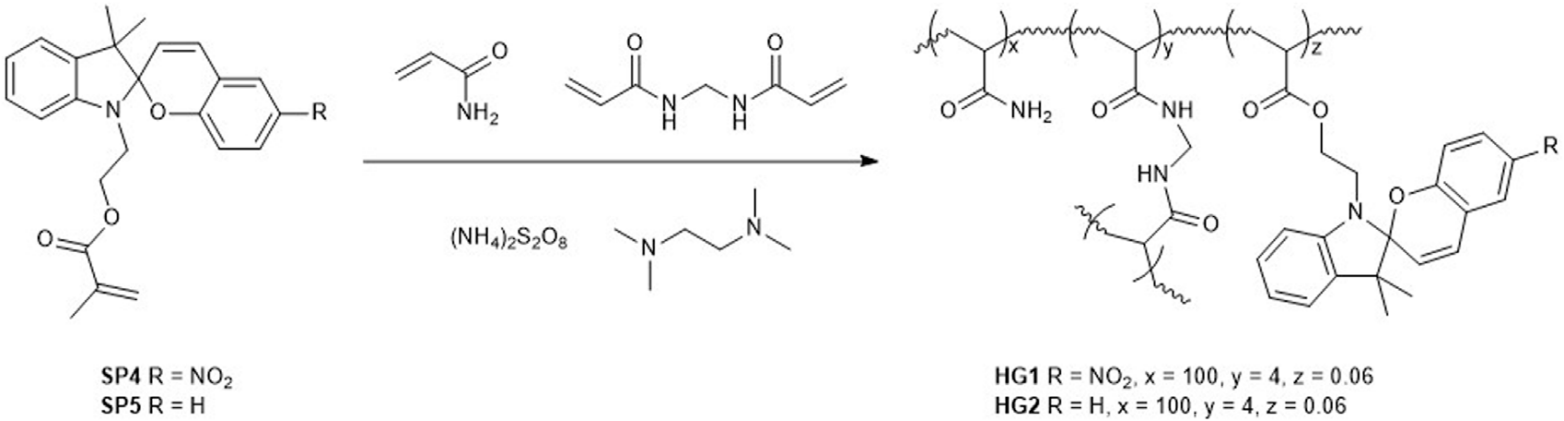
Synthesis of SP-loaded acrylamide hydrogels HG1 and HG2 using spiropyran-acrylate conjugates SP1 and SP2.

To transfer the acidochromic behavior of SPs to solid materials that could be applied to CO_2_ detection directly from the gas phase, we devised the utilization of SP-loaded hydrogels (HGs). Because of the similar acidochromic properties of SP2 and SP3, only the use of SP2 was considered for the preparation of HGs with high pK_a_ (McH), as it is more synthetically accessible than SP3. In addition, SP1-based HGs were also targeted as low pK_a_ (McH) materials. In all these cases, we decided to covalently anchor SPs to the hydrogel matrix, thus aiming to prevent their migration and loss during manipulation and use. For this, we followed reported procedures for the incorporation of SPs in acrylamide HGs (Li et al., 2020), which required the previous synthesis of two spiropyran-acrylate conjugates: SP4 (He et al., 2017) and SP5 (Li et al., 2020), which present the same spiropyran dyes as SP1 and SP2, respectively (Scheme 2 and Supplementary Figures S13, S14). These compounds were then added to DMSO:water 4:1 solutions of acrylamide and bisacrylamide monomers, which were subjected to radical polymerization in glass containers. The resulting gels were subsequently treated with MeOH to remove unreacted species and immersed in water to obtain the target hydrogels HG1 (SP4) and HG2 (SP5) with a thickness of ∼ 5 mm, a swelling ratio of ca. 27 and a SP content of ∼ 1.2 10^−6^ wt.% (Scheme 2). As reported for other acrylamide HGs (Gombert et al., 2020), scanning electron microscopy analysis of HG1 and HG2 after freeze-drying revealed their hollow pore structure (Supplementary Figure S15A). As for their mechanical properties, they were characterized by dynamic mechanical analysis (DMA), which provided very similar values of storage modulus (G′) and loss modulus (G″) for both types of gels: G’ ∼ 700 Pa and G” ∼ 15 Pa (Supplementary Figure S15B). The large G”/G’ ratio determined (∼50) is consistent with the chemical gel-like nature of HG1 and HG2, whose covalent cross-linking polymer phase confers a predominant elastic behavior over a rather large linear viscoelastic range (up to 10% strain; Supplementary Figure S15C).

**FIGURE 4:**
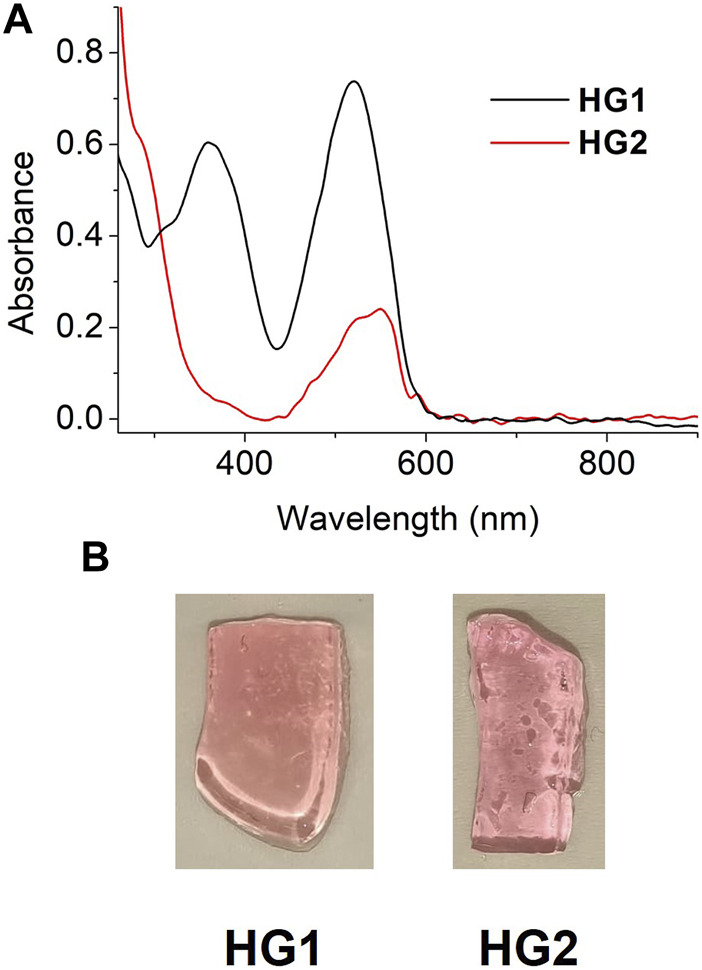
**(A)** UV-vis absorption spectra of freshly prepared HG1 and HG2 under ambient conditions. **(B)** Photographs of pieces of the hydrogels measured in **(A)**.

Thanks to the low spiropyran loading, HG1 and HG2 were sufficiently translucent as to be characterized by UV-vis absorption spectroscopy in transmission mode. Figure 4A shows the UV-vis absorption spectra of freshly prepared HG1 and HG2 at room temperature, which resemble those measured for SP1 and SP2 in water at pH = 7. In both cases, we observed the presence of a clear absorption band at λ_max_ ∼ 520 (HG1) and 550 nm (HG2), which can be unambiguously ascribed to the fraction of spiropyran molecules in the hydrogels that are in their Mc state. As a result, both materials presented a pink coloration similar to that of diluted aqueous solutions of SP1 and SP2 at neutral pH (Figure 4B). In addition, HG1 and HG2 preserved the photochromic properties of their constituting spiropyrans, as they nearly turned colorless upon irradiation with green light due to reversible light-induced Mc → Sp conversion (see Supplementary Figure S16). However, this behavior was observed to be lost after storage of the hydrogels for several weeks under ambient conditions, whose color evolved to lighter pink. Most probably, this was due to SP hydrolysis inside the hydrogels, which is known to take place in neutral aqueous media for the unprotonated Mc form (Hammarson et al., 2013; Berton et al., 2020; Wimberger et al., 2021a).

**FIGURE 5:**
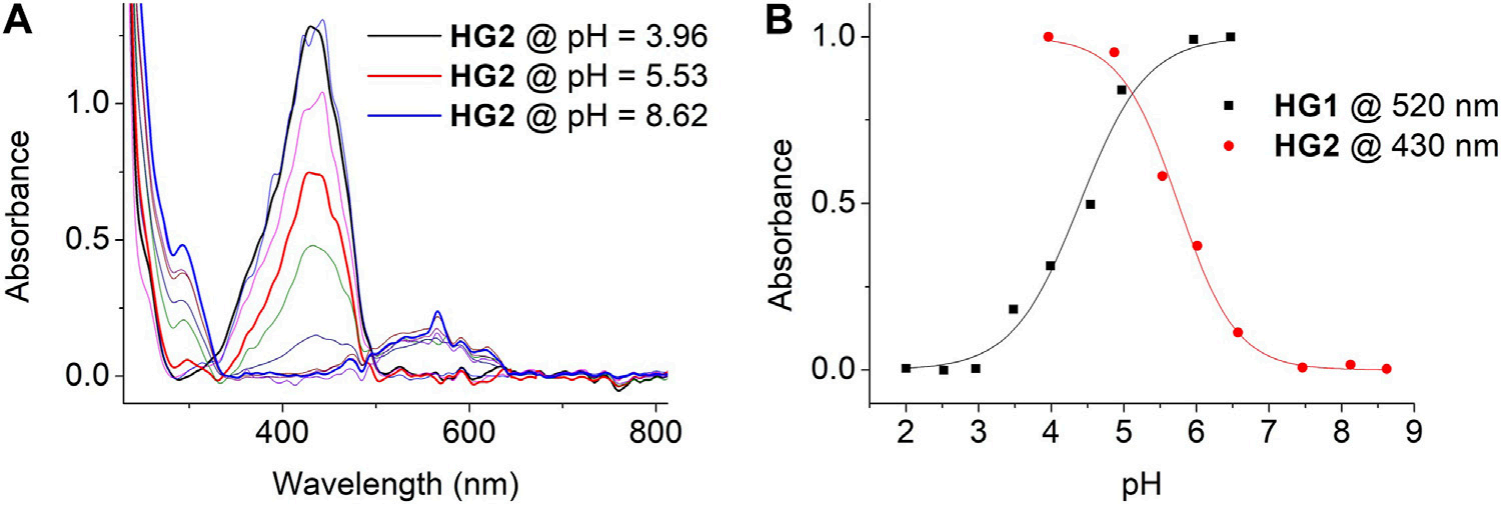
**(A)** Variation of the UV-vis absorption of HG2. Measurements were conducted by immersing HG2 in independent buffer solutions of pH = 3.96, 4.45, 4.87, 5.53, 6.01, 6.57, 7.04, 7.46, 8.12 and 8.62. **(B)** pH-induced variation of the absorbance of HG1 and HG2 in aqueous media at λ = 520 (HG1) and 430 nm (HG2). Symbols correspond to experimental data, while lines are fits to Eq. 1 for pK_a_ (McH) determination (see Section 4.4).

Next, we evaluated the acidochromic behavior of HG1 and HG2 when immersed in aqueous buffers of different pH. Similar responses to those registered for SP1 and SP2 in free solution were registered for the hydrogels, which were mainly characterized by the emergence of the typical McH absorption band at λ_max_ ∼ 430 nm upon acidification (Figure 5A and Supplementary Figure S17) - i.e., they turned yellow at sufficiently acid pH values. Importantly, we could use this data to determine the pK_a_ (McH) in the dark of the spiropyrans loaded in HG1 and HG2: pK_a_ (McH) = 4.40 and 5.75, respectively (Figure 5B). These values are slightly lower than those found for SP1 and SP2 in water, which must be attributed to the absence of the negatively charged sulfonate group for the spiropyrans anchored to the hydrogels. In particular, it has been demonstrated for a series of *N*-substituted SPs that such group stabilizes the nearby positive charge of the indolium ring of Mc and McH, thus increasing pK_a_ (McH) in the dark (Wimberger et al., 2021a). In spite of this, the SP acidity constants of the two hydrogels prepared still lay on the desired ranges to achieve selective CO_2_ detection: (a) pK_a_ (McH) < 4.5 for HG1, which should make it nearly insensitive to CO_2_ exposure, and (b) pK_a_ (McH) ∼ 5-6 for HG2, which should allow for CO_2_-induced colorimetry with optimal sensitivity. Actually, as the pH detection window of acidochromic compounds typically lies ∼ 1.5 units around their pK_a_ value (Steinegger et al., 2020), HG2 should be sensitive to changes within the pH ∼ 4.25–7.25 interval. This essentially matches the dynamic range needed for CO_2_ determination in water at room temperature and atmospheric pressure, which spans from pH ∼ 4.0 to 7.0 (Haghi et al., 2017).

### 2.3 CO_2_ absorption and detection with spiropyran-based hydrogels

**FIGURE 6:**
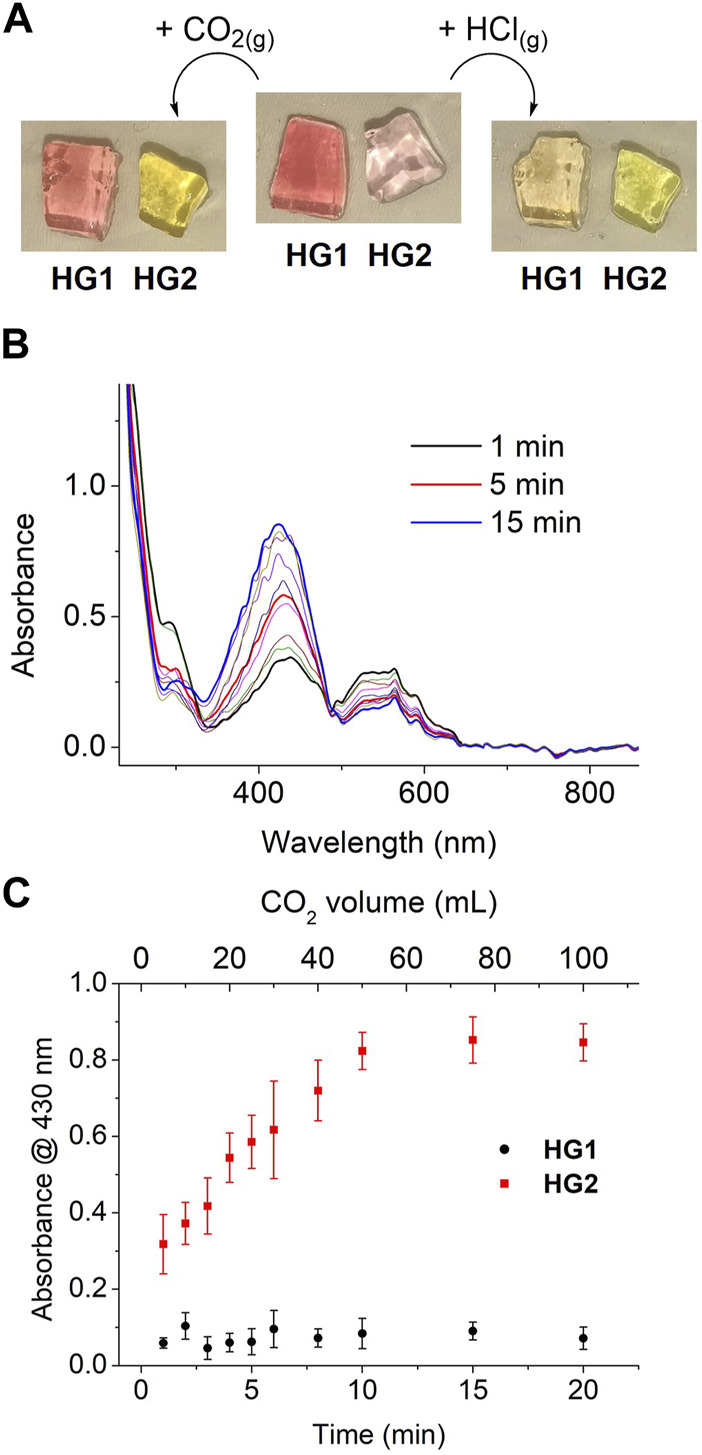
**(A)** Images of freshly prepared HG1 and HG2 before and after exposure to gaseous CO_2_ or HCl fluxes until no more changes in color were observed. **(B)** Variation of the UV-vis absorption spectrum of HG2 when subjected to different times of exposure to a regular flux of gaseous CO_2_ (5 mL min^-1^). **(C)** Variation of the absorbance of HG1 and HG2 at λ = 430 nm when subjected to different times of exposure to a regular flux of gaseous CO_2_ (5 mL min^-1^). Error bars show the standard errors of the mean for 5 replicates measured in different hydrogels. For each exposure time, the concentration of CO_2_ absorbed within the gel was estimated to be 0.10, 0.15, 0.23, 0.70, 1.04, 1.44, 5.49, 116.34, 485.17 and 2023.29 mg L^-1^.

To demonstrate that the distinct acidochromic properties of HG1 and HG2 allow selective detection of CO_2_, we first investigated their colorimetric behavior when fully saturated with fluxes of two different acid gases: CO_2_ and HCl. As observed in Figure 6A, only HG2 was found to respond to CO_2_ exposure and suffer the expected pink-to-yellow color change - i.e., the protonation of its spiropyran moieties into the yellow-colored McH form. By contrast, the red-pink color of HG1 remained almost unaltered, which indicates that no (or minor) spiropyran protonation took place due to its lower pK_a_ (McH). As expected, a different situation was encountered upon treatment with a stronger and more water-soluble gas such as HCl. In this case, both HG1 and HG2 underwent the color variation associated to McH formation, which demonstrates that lower pH values were reached within the hydrogels (Figure 6A). Interestingly, these results prove the viability of our strategy to discriminate between the acidochromic responses caused by different acids. In addition, they demonstrate that SP-based hydrogels permit direct colorimetric detection of gaseous CO_2_ without the need of any prior treatment, thus enhancing the measurement conditions described in previous reports based on polymer fibers functionalized with nitrospiropyran derivatives (Shang et al., 2017; Dekhordi et al., 2021). Moreover, it must also be noted that, in opposition to these precedents, the colorimetric response to CO_2_ of our hydrogels does agree with the reported behavior of SPs upon protonation in aqueous solution.

Encouraged by these positive results, we further investigated the chromic detection of CO_2_ with HG1 and HG2. For this, their UV-vis absorption spectra were measured upon exposure to controlled amounts of CO_2_ (from 0 to 100 mL at a regular flux of 5 mL min^-1^). Importantly, no differences were observed when these experiments were conducted under ambient light or in the dark, thus indicating that the acidochromic responses of the gels are not affected by low intensity illumination. This, in combination with the fact that the spiropyran molecules confined in HG1 and HG2 are not exposed to other chemical stimuli that could promote color changes (e.g., metal ions, solvent variation), should make their chromic behavior essentially report on CO_2_ interaction–i.e., on CO_2_ absorption into the gels and subsequent acidification of their aqueous liquid phase. In fact, steady spectral variations were registered for HG2 in these experiments that are compatible with CO_2_-induced continuous spiropyran protonation: the absorption band of Mc at λ_max_ ∼ 550 nm progressively disappeared, whereas the absorbance of McH at λ_max_ ∼ 430 nm concomitantly raised (Figure 6B). By contrast, negligible absorption changes were measured for HG1 under the same conditions. This is clearly illustrated by Figure 6C, where the variation of the absorbance at λ_max_ (McH) with the flowed CO_2_ volume is plotted for both HG1 and HG2. Aside from the large differential response of both hydrogels to CO_2_, two additional conclusions can be inferred from this figure. First, it must be noted that CO_2_-induced color changes in HG2 occurred very rapidly (less than 1 min), thus demonstrating the capacity of our system to quickly sense CO_2_ concentration changes in the surrounding atmosphere. However, saturation of the colorimetric response required large CO_2_ volumes, probably because of the time needed for the gas to diffuse throughout the whole system and reach all the embedded SP molecules (∼ several minutes). Second, a rather linear response with CO_2_ exposure volume was found, which could be used to conduct quantitative CO_2_ measurements. Actually, based on our previous determination of the pH-dependent variation of the absorption spectra of HG2, the CO_2_-induced color changes measured for this sample could be correlated to the amount of CO_2_ dissolved within the gel (Supplementary Figure S18). According to this data, we could estimate that the CO_2_ detection range of HG2 spans from 0 to 500 mg L^-1^ with a detection limit of 0.10 mg L^-1^.

**FIGURE 7:**
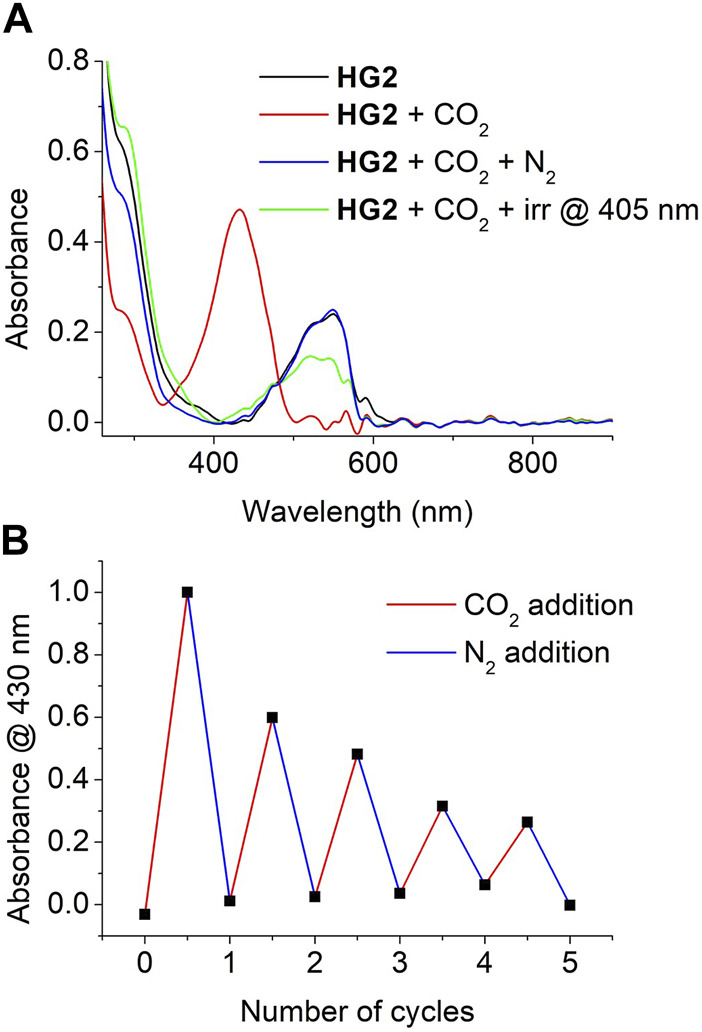
**(A)** UV-vis absorption of HG2 measured at the following conditions: (a) after preparation; (b) after saturation with gaseous CO_2_; (c) after exposure of the CO_2_-saturated hydrogel to a mild flux of N_2_ for 40 min; and (d) after irradiation of the CO_2_-saturated hydrogel at 405 nm for 5 min **(B)** Variation of the absorbance of HG2 at λ = 430 nm when subjected to 5 consecutive cycles of CO_2_ saturation and N_2_-induced CO_2_ desorption.

Finally, we explored the reversibility of the CO_2_-promoted colorimetric response of HG2. In a first attempt, we just exposed a CO_2_-saturated hydrogel to ambient air, which eventually resulted in CO_2_ release and the recovery of its initial UV-vis absorption spectrum with no presence of McH absorbance. However, this process was observed to be slow, as it took about 120 min to be completed. Alternatively, we aimed to accelerate CO_2_ desorption by exposing HG2 to a mild N_2_ flux. As observed in Figure 7A, this also allowed the absorbance of the initial CO_2_-free hydrogel to be recovered, though it still required rather long times (∼40 min). As a result, nonnegligible loss of the water content of the hydrogel was observed to take place during CO_2_ desorption, which resulted in a visible change in the rugosity and elasticity of the material that could not be fully recovered by subsequent immersion in water. Unfortunately, this situation was aggravated when HG2 was subjected to consecutive cycles of CO_2_ absorption (∼20 min) and N_2_-induced CO_2_ desorption (∼40 min), which had a detrimental effect on the amplitude of the colorimetric response obtained (Figure 7B). In spite of this, clear CO_2_-induced pink-to-yellow color changes could still be registered for HG2 after 5 cycles of reuse. Surprisingly, we also observed that the recovery of HG2 after CO_2_ detection could be largely accelerated upon irradiation with high intensity light-emitting diodes (LEDs). In particular, illumination at 405 nm of the McH species in the hydrogel after CO_2_ absorption led to a complete loss of their absorption signal at λ_max_ ∼ 430 nm after just 5 min. Importantly, the Mc absorbance at λ_max_ ∼ 550 nm was simultaneously recovered in a large extent, thus confirming that McH illumination did not just lead to *cis*-McH formation in our hydrogel by photoisomerization but also to deprotonation to recover the initial Sp-Mc mixture. Therefore, illumination allows faster recyclability of HG2 for subsequent reuse.

## 3 Conclusion

In this work we reported a new strategy for exploiting the acidochromic properties of spiropyrans to accomplish selective colorimetric detection of gaseous CO_2_ with solid materials. For this, we first demonstrated that chemical tailoring of the acidity constant of SPs allows discriminating the color changes caused in aqueous media by CO_2_ from other acid gases. Thus, only SPs whose protonated merocyanine form shows a sufficiently high pK_a_ in the dark (pK_a_ (McH) ∼ 5–6) led to ample colorimetric responses when exposed to CO_2_ in water solution. By contrast, more water-soluble and stronger acids such as HCl also triggered similar color variations for SPs with lower pK_a_ (McH) (pK_a_ (McH) < 4.5). To transfer this behavior to the solid state, we prepared hydrogels functionalized with SPs of distinct acid-base properties, whose pK_a_ (McH) values were proven to be essentially preserved inside these materials. As a result, differential colorimetric responses could be promoted for these hydrogels when treated with CO_2_ relative to HCl, thus allowing selective optical detection of carbon dioxide. In addition, we demonstrated that the CO_2_-induced color changes in the hydrogels are fast, quantifiable and reversible, while they can be directly measured at the gas-solid interface without the need of immersion or pretreatment with water. In light of these properties, our spiropyran-based hydrogels appear as promising systems for the colorimetric detection of CO_2_ in a wide range of applications.

## 4 Materials and methods

### 4.1 Materials and characterization methods

Commercial reagents were used as received. Anhydrous THF was used after column drying in a solvent dispenser from Innovative technology (PureSolv-MD-2). Acetonitrile was dried with molecular sieves, 3 A beads, 4-8 mesh from Sigma-Aldrich. Reactions were monitored by analytical thin-layer chromatography (TLC), using silica gel 60 F254 precoated aluminium plates (0.20 mm thickness), and development was made using a UV lamp at 254 nm. Flash column chromatography was performed using silica gel (230–400 mesh).

NMR spectra were recorded on Bruker DPX360 (360 MHz for ^1^H NMR), Bruker Ascend 300 MHz (300 MHz for ^1^H NMR) and Bruker Ascend 400 MHz (400 MHz for ^1^H NMR) spectrometers, using the residual peaks of the deuterated solvents as a reference. IR-ATR spectra were recorded in a Bruker Tensor 27 Golden Gate spectrometer with a diamond tip. Mass spectra were recorded in a Bruker Esquire 3,000+ spectrometer using ESI and APCI. UV-Vis absorption spectra were recorded on an Agilent HP 8453 spectrophotometer using HPLC quality solvents and 1 cm quartz cuvettes. To photoisomerize compounds, LEDs at 405 nm and 532 nm and UV lamps at 312 nm and 365 nm were used. pH was measured using a Hach sensION mm340 pHmeter. For CO_2_ capture tests, a Bronkhorst EL-FLOW flow meter was used and controlled by FlowDDE and FlowView software. Scanning electron microscopy analysis of the hydrogels was conducted with a FEI Quanta 650 ESEM microscope applying a 5 kV voltage. Prior to SEM imaging, hydrogels were freeze-dried and metalized with a layer of about 5 nm of Pt. Dynamic mechanical analysis of HG1 and HG2 was conducted by means of strain sweep measurements using a Discovery HR-2 rheometer at room temperature (frequency = 1 Hz). 5 samples of each type of gel were analyzed independently to determine the average storage and loss moduli.

### 4.2 Synthesis of spiropyran derivatives SP1-SP5

#### 4.2.1 Synthesis of SP1, SP2, SP4 and SP5

Spiropyrans SP1 (Liu et al., 2019), SP2 (Shi et al., 2011), SP4 (He et al., 2017) and SP5 (Li et al., 2020) were prepared according to reported procedures. SP1 and SP2 were isolated as the corresponding triethylammonium salts. For all of these compounds (and SP3), NMR characterization was performed in nonaqueous solvents, where they exist as the colorless spiranic form.

SP1: ^1^H NMR (360 MHz, CD_3_OD) δ = 8.09 (d, *J* = 2.8 Hz, 1H), 8.02 (dd, *J* = 9.0, 2.7 Hz, 1H), 7.13 (td, *J* = 7.7, 1.3 Hz, 1H), 7.10–7.03 (m, 2H), 6.84–6.75 (m, 2H), 6.70 (d, *J* = 7.8 Hz, 1H), 6.02 (d, *J* = 10.4 Hz, 1H), 3.43–3.26 (m, 2H), 3.20 (q, *J* = 7.3 Hz, 6H), 2.92–2.74 (m, 2H), 2.19–2.02 (m, 2H), 1.30 (t, *J* = 7.3 Hz, 9H), 1.27 (s, 3H), 1.18 (s, 3H) ppm.

SP2: ^1^H NMR (360 MHz, CD_3_OD) δ = 7.04–6.91 (m, 4H), 6.83 (d, *J* = 10.2 Hz, 1H), 6.75–6.64 (m, 2H), 6.58–6.50 (m, 2H), 5.70 (d, *J* = 10.3 Hz, 1H), 3.32–3.08 (m, 2H), 2.97 (q, *J* = 7.2 Hz, 6H), 2.83–2.64 (m, 2H), 2.11–1.90 (m, 2H), 1.17 (s, 3H), 1.16 (t, *J* = 7.3 Hz, 9H), 1.06 (s, 3H) ppm.

SP4: ^1^H NMR (300 MHz, CDCl_3_): δ = 8.11–7.97 (m, 2H), 7.23 (td, *J*_*1*_ = 7.7 Hz, *J*_*2*_ = 1.3 Hz, 1H), 7.12 (ddd, *J*_*1*_ = 7.3 Hz, *J*_*2*_ = 1.3 Hz, *J*_*3*_ = 0.5 Hz, 1H), 6.98–6.86 (m, 2H), 6.77 (dt, *J*_*1*_ = 8.3 Hz, *J*_*2*_ = 0.8 Hz, 1H), 6.75–6.70 (m, 1H), 6.09 (dq, *J*_*1*_ = 1.6 Hz, *J*_*2*_ = 1.0 Hz, 1H), 5.90 (d, *J* = 10.3 Hz, 1H), 5.59 (q_t_, *J* = 1.6 Hz, 1H), 4.32 (dd, *J*_*1*_ = 6.6 Hz, *J*_*2*_ = 6.0 Hz, 2H), 3.58 (dt, *J*_*1*_ = 15.1 Hz, *J*_*2*_ = 6.6 Hz, 1H), 3.45 (dt, *J*_*1*_ = 15.1 Hz, *J*_*2*_ = 6.0 Hz, 1H), 1.94 (dd, *J*_*1*_ = 1.6 Hz, *J*_*2*_ = 1.0 Hz, 3H), 1.30 (s, 3H), 1.19 (s, 3H) ppm.

SP5: ^1^H NMR (300 MHz, CDCl_3_): δ = 7.21 (tt, *J*_*1*_ = 7.7 Hz, *J*_*2*_ = 1.3 Hz, 1H), 7.17–7.04 (m, 3H), 6.92–6.82 (m, 3H), 6.71 (dd, *J*_*1*_ = 7.9 Hz, *J*_*2*_ = 2.6 Hz, 2H), 6.11 (dq, *J*_*1*_ = 1.6 Hz, *J*_*2*_ = 1.0 Hz, 1H), 5.72 (d, *J* = 10.1 Hz, 1H), 5.58 (q_t_, *J* = 1.6 Hz, 1H), 4.33 (t, *J* = 6.4 Hz, 2H), 3.69–3.58 (m, 1H), 3.43 (dt, *J*_*1*_ = 15.1 Hz, *J*_*2*_ = 6.2 Hz, 1H), 1.95 (s, 2H), 1.33 (s, 3H), 1.18 (s, 3H) ppm.

#### 4.2.2 Synthesis of SP3

##### 4.2.2.1 Synthesis of 2-bromo-3,5-dimethoxybenzaldehyde (2)

A mixture of 2.012 g of 3,5-dimethoxybenzaldehyde (12.11 mmol, **1**) and 2.222 g of *N*-bromosuccinimide (12.48 mmol) in 20 mL of anhydrous acetonitrile was stirred at room temperature overnight. 60 mL of aqueous NaOH were added and the mixture was extracted with ethyl acetate (30 mL) three times. The combined organic phases were dried over anhydrous Na_2_SO_4_ and the solvent was removed under vacuum to yield 2.849 g of **2** (11.62 mmol; 96% yield) as a pale powder. Its ^1^H-NMR spectrum was in accordance with bibliographical data (Liao et al., 2012). ^1^H NMR (300 MHz, CDCl_3_): δ = 10.44 (s, 1H), 7.07 (d, *J* = 2.8 Hz, 1H), 6.74 (d, *J* = 2.8 Hz, 1H), 3.94 (s, 3H), 3.88 (s, 3H) ppm.

##### 4.2.2.2 Synthesis of 2-(2-bromo-3,5-dimethoxyphenyl)-1,3-dioxolane (3)

In a two necked bottom flask equipped with a Dean-Stark apparatus, a solution of 3.063 g of **2** (12.50 mmol), 6.6 mL of ethylene glycol (118.3 mmol) and 0.157 g of *p*-toluene sulfonic acid (0.82 mmol) in 120 mL of toluene was refluxed overnight. Next, the reaction was poured into 100 mL of a NaOH solution 3 M and the mixture was extracted twice with 100 mL of CH_2_Cl_2_. Once the organic phase was dried with anhydrous Na_2_SO_4_ and the solvent removed under vacuum, purification was conducted through flash column chromatography with chloroform to yield 3.082 g of **3** (10.66 mmol; 85% yield). Its ^1^H-NMR spectrum was in accordance with bibliographical data (Huang et al., 2011). ^1^H NMR (300 MHz, CDCl_3_): δ = 6.82 (d, *J* = 2.8 Hz, 1H), 6.53 (d, *J* = 2.8 Hz, 1H), 6.15 (s, 1H), 4.21–4.14 (m, 2H), 4.14–4.06 (m, 2H), 3.90 (s, 3H), 3.85 (s, 3H) ppm.

##### 4.2.2.3 Synthesis of 2-hydroxy-3,5-dimethoxybenzaldehyde (4)

A solution of 1.030 g of **3** (3.56 mmol) in 25 mL of anhydrous THF was purged with argon and cooled in an acetone bath at −78°C. 2.4 mL of a 1.6 M solution of *n*-BuLi (3.84 mmol) in hexane were added and the solution was stirred for 5 min at −78°C. Then, 2 mL of B(OBu)_3_ (7.41 mmol) were added, the cold bath was removed, and the solution was stirred for 1 h. Afterwards, 10 mL of water and 4 mL of 30% H_2_O_2_ were added, and the solution was stirred for 30 min. The reaction was quenched in a cold bath by addition of a solution of 10.076 g of Na_2_S_2_O_3_ in 15 mL of water, and then the crude was then extracted with Et_2_O, the organic phase was dried with anhydrous Na_2_SO_4_, and the solvent removed under vacuum. The resulting oil was redissolved with 40 mL of acetone and 40 mL of HCl 1M, and the solution was stirred for 2 h. The crude was first extracted with AcOEt. Then, the organic phase was extracted with NaOH 1M and concentrated HCl was added to the resulting aqueous phase in a cold bath until some turbulence appeared (acid pH). Finally, the acid aqueous phase was extracted with CHCl_3_ and the solvent removed under vacuum. The solid obtained was further purified through flash column chromatography with chloroform to yield 0.492 g of **4** (2.70 mmol; 75% yield) as a yellow solid. Its ^1^H-NMR spectrum was in accordance with bibliographical data (Liao et al., 2012). ^1^H NMR (300 MHz, CDCl_3_): δ = 10.70 (s, 1H), 9.91 (s, 1H), 6.77 (d, *J* = 2.8 Hz, 1H), 6.61 (d, *J* = 2.8 Hz, 1H), 3.92 (s, 3H), 3.85 (s, 3H) ppm.

##### 4.2.2.4 Synthesis of 3-(2,3,3-trimethyl-3H-indol-1-ium-1-yl)propane-1-sulfonate (5)

A solution of 0.992 g of 2,3,3-trimethylindolenine (6.23 mmol) and 0.835 g of 1,3-propanesultone (6.84 mmol) in 5 mL of toluene was refluxed under argon atmosphere for 12 h. The crude was cooled in an ice bath and the precipitated was filtered and then dissolved in water. The resulting solution was extracted with diethyl ether twice and the solvent removed under vacuum to yield 1.203 g of **5** (4.28 mmol; 69% yield). Its ^1^H-NMR spectrum was in accordance with bibliographical data (Shi et al., 2011). ^1^H NMR (360 MHz, CDCl_3_): δ = 7.96 (d, *J* = 7.0 Hz, 1H), 7.62-7-50 (m, 3H), 5.00 (t, *J* = 7.8 Hz, 2H), 3.08–3.02 (m, 5H), 2.40 (br, 2H), 1.61 (s, 6H) ppm.

##### 4.2.2.5 Synthesis of SP3

SP3 was prepared following a procedure reported for the synthesis of other spiropyrans (Pargaonkar et al., 2018). 0.329 g of **4** (1.80 mmol) and 0.502 g of **5** (1.80 mmol) were dissolved in 2 mL of a 40% solution of choline hydroxide in water and were heated at 80°C for 1 h. The solvent was removed under vacuum and the residue was purified through flash column chromatography with CHCl_3_:MeOH 9:1 to yield a red solid, which was dissolved in MeOH, Et_3_N was added until the solution was clear and the solvent was removed under vacuum to yield 0.383 g of the triethylammonium salt of SP3 (0.70 mmol; 39% yield) as a dark solid. ^1^H NMR (300 MHz, DMSO-*d*_6_) δ = 7.11–7.01 (m, 2H), 6.89 (d, *J* = 10.2 Hz, 1H), 6.71 (td, *J* = 7.4, 0.9 Hz, 1H), 6.60 (d, *J* = 7.7 Hz, 1H), 6.42 (d, *J* = 2.8 Hz, 1H), 6.37 (d, *J* = 2.8 Hz, 1H), 5.73 (d, *J* = 10.2 Hz, 1H), 3.69 (s, 3H), 3.61 (s, 3H), 3.25–3.08 (m, 2H), 2.85 (br, 6H), 2.45–2.33 (m, 2H), 1.83 (q_t_, *J* = 7.6 Hz, 2H), 1.16 (s, 3H), 1.12–1.01 (m, 12H) ppm; ^13^C NMR (75 MHz, DMSO-*d*_6_) δ = 153.15, 147.85, 147.59, 137.49, 136.49, 129.62, 127.77, 121.95, 121.40, 119.20, 118.53, 106.62, 104.11, 102.61, 101.45, 55.95, 55.82, 52.15, 49.73, 46.18, 42.83, 26.35, 25.44, 20.09, 10.21 ppm; IR (ATR, cm^-1^): 2,966, 2,687, 2,363, 2,342, 1,587, 1,481, 1,393, 1,355, 1,288, 1,240, 1,213, 1,198, 1,158, 1,109, 1,017, 981, 948, 827, 808, 746, 681, 655; HRMS (ESI) m/z: [M+H+Na]^+^ calcd for C_23_H_27_NNaO_6_S^+^ 468.1451; found 468.1434.

### 4.3 Synthesis of SP-based hydrogels HG1-HG2

The synthesis of spiropyran-based hydrogels was carried according to a reported procedure (Li et al., 2020). Hydrogels were prepared by dissolving 142.2 mg (2 mmols) of acrylamide, 12.4 mg (80 μmol) of *N*,*N*-methylenebisacrylamide, 1.2 μmol of each polymerizable spiropyran (SP4 or SP5) and 9.1 mg (40 μmol) of ammonium persulfate in 2.2 mL of a 4:1 DMSO/water mixture. Then, 9 μL (60 μmol) of tetramethylethylenediamine were added at once to start the polymerization at room temperature for 2 h. The resultant gel was soaked in MeOH and then water overnight to obtain the final gel which was cut into small pieces for optical characterization.

### 4.4 Characterization of the acidochromic properties of SP1-SP3 and HG1-HG2

The pH-induced variation of the UV-vis absorption spectra of SP1-3 were measured in buffer solutions using 1-cm quartz cuvettes. Buffer solutions were prepared by adding HCl (35 wt.%) to a K_3_PO_4_ (100 mM) solution while measuring the pH of the final sample. Then, 2 mL of the corresponding buffer were mixed with 20 μL of a stock solution of the SP of interest (c ∼ 10^–3^ M) in a 1-cm quartz cuvette; then, the resulting mixture was left to equilibrate for different times before the final absorption spectrum in the dark was registered (up to 2 h for SP1, 5–15 min for SP2 and < 1 min for SP3). To investigate the light effect on the acidochromic behavior, irradiation at 405 nm for 5 min was conducted before measuring the absorbance spectrum.

For HG1-2, pH-dependent UV-vis absorption measurements were performed in air using a custom-made metallic T-shaped cuvette where a piece of the gels was sandwiched between two metal plates having a 0.5 cm-in-diameter opening to allow the light beam to go through the sample. Before their measurement, the gels were submerged in 5 mL of the buffer solution of interest in a glass vial and left to equilibrate for 90 min.

In order to determine the pK_a_ (McH) values of SP1-SP3 and HG1-HG2, their absorbance at selected wavelengths (505 nm for SP1, 425 nm for SP2, 585 nm for SP3, 520 nm for HG1, and 420 nm for HG2) were plotted against the pH of the buffer solutions used and the resulting plots were fitted to Equation 1 (Berton et al., 2020; Wimberger et al., 2021a).

$$A_{eq}=A_{bas}+\frac{A_{ac}-A_{bas}}{1+\exp\left(\frac{pH-{pK}_{a}\left(McH\right)}{P}\right)}$$ (1)

In this equation, A_eq_ is the absorbance measured at each pH, A_bas_ is the absorbance at the most basic pH, A_ac_ is the absorbance at the most acidic pH and P is a parameter of the fitting.

### 4.5 Acid gas detection tests with HG1-HG2

To investigate the colorimetric response of HG1-HG2 to different acid gases, pieces of the gels were introduced in closed vials where the gases of interest were injected. CO_2(g)_ (>99.995% purity) was introduced using to different ways: (a) in a controlled manner with a flow meter (5 mL min^-1^) for defined times, or (b) injecting a balloon filled with CO_2_. HCl_(g)_ was introduced by bubbling a solution of concentrated HCl with N_2_. When full saturation of the hydrogels with CO_2(g)_ or HCl_(g)_ was desired, samples were left to equilibrate for 30 min so that gases could completely diffuse inside the gels. In all the cases, UV-vis absorption measurements were conducted in air as described in Section 4.4. To revert back the acidochromic response from HG1-HG2, the gels were left in open air for about 120 min, exposed to a N_2_ atmosphere in a closed vial for about 40 min, or irradiated with an LED at 405 nm (51 mW cm^-2^) for 5 min. For HG2, the CO_2_ concentration absorbed within the gel for each exposure time was estimated from: (a) the pH dependence of the absorption spectra of HG2; (b) the acidity constants of CO_2_ (pK_a1_ = 6.36) and HCO_3_^−^ (pK_a2_ = 10.25) in pure water at 25°C and atmospheric pressure and the autoionization constant of water at these conditions (pK_w_ = 14).

## Data Availability

The raw and processed data required to reproduce these findings is available in the CORA.RDR, Research Data Repository (https://dataverse.csuc.cat/): https://doi.org/10.34810/data727 (accessed on 16 May 2023).
