# Supplementary material for: Spiropyran-based chromic hydrogels for CO2 absorption and detection
Source: Front Chem. 2023 May 23;11:1176661. doi: 10.3389/fchem.2023.1176661 (PMC10242082; doi:10.3389/fchem.2023.1176661)
Supplement: Supplementary file 1 [file DataSheet1.pdf]

Supplementary Material

Spiropyran-Based Chromic Hydrogels for CO_2 Absorption and Detection

Arnau Marco, Gonzalo Guirado, Rosa María Sebastián*, Jordi Hernando*

Departament de Química, Universitat Autònoma de Barcelona, Cerdanyola del Vallès, Spain

*** Correspondence:**
Corresponding Author
rosamaria.sebastian@uab.cat; jordi.hernando@uab.cat

TABLE OF CONTENTS

1. Supplementary figures, schemes and tables 2

2. References 14

1. Supplementary figures, schemes and tables

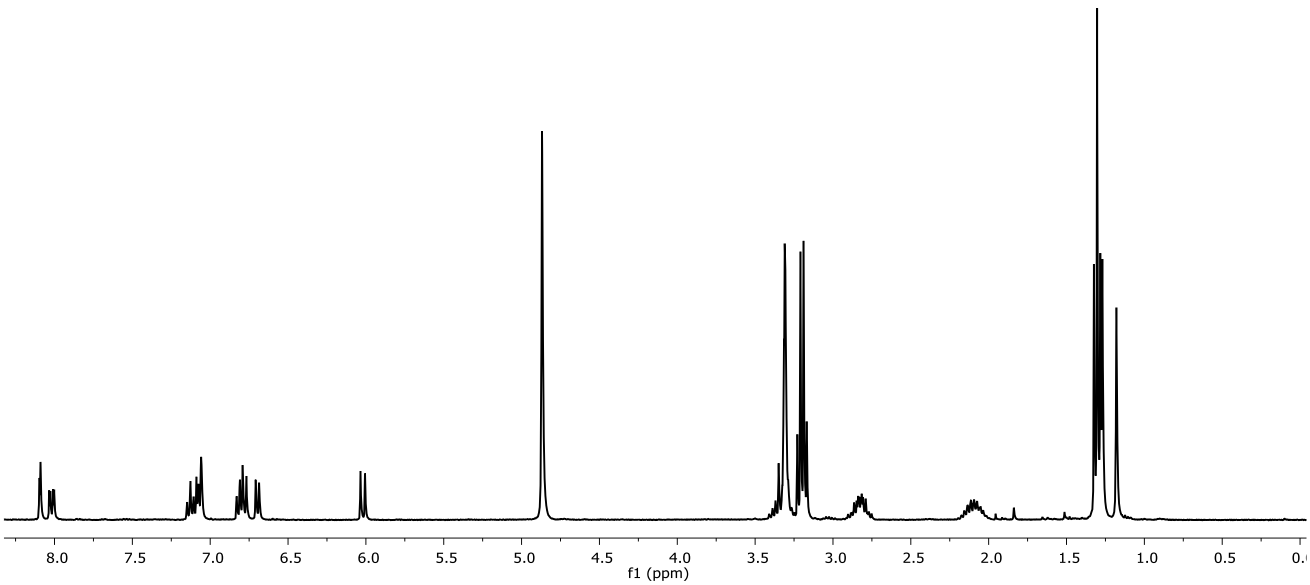

Supplementary Figure S1. ^{1}H NMR spectrum (360 MHz, CD_3OD) of **SP1**.

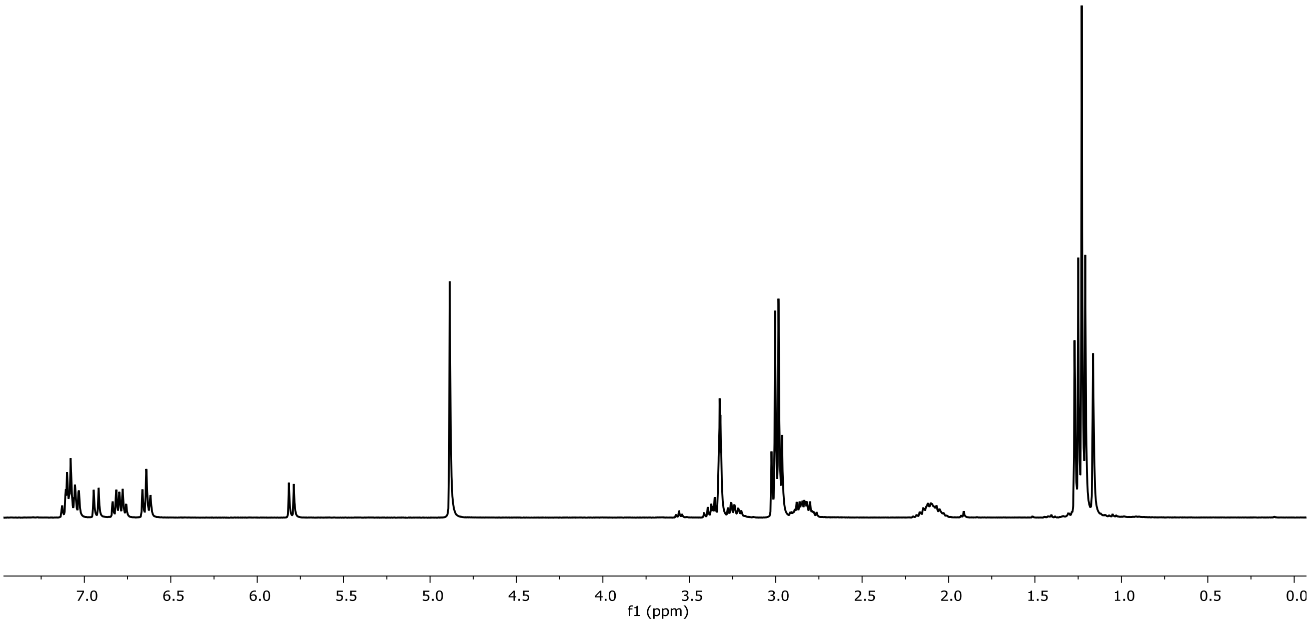

Supplementary Figure S2. ^{1}H NMR spectrum (360 MHz, CD_3OD) of **SP2**.

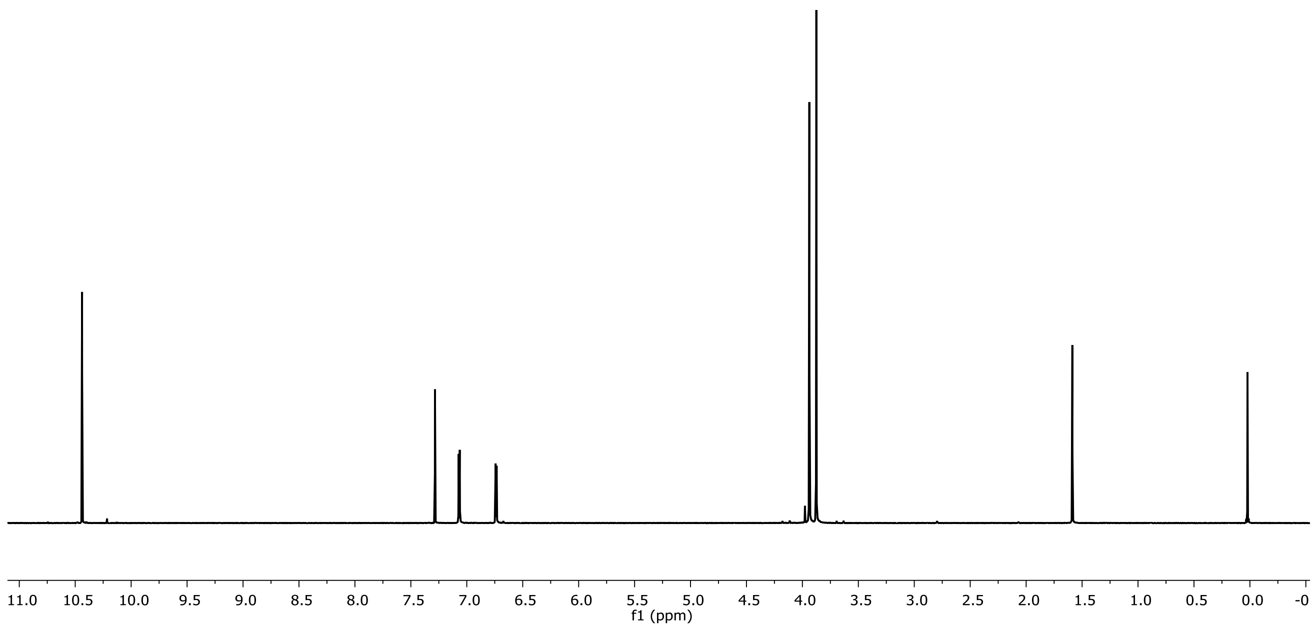

Supplementary Figure S3. ^{1}H NMR spectrum (300 MHz, $CDCl_3$) of **2**.

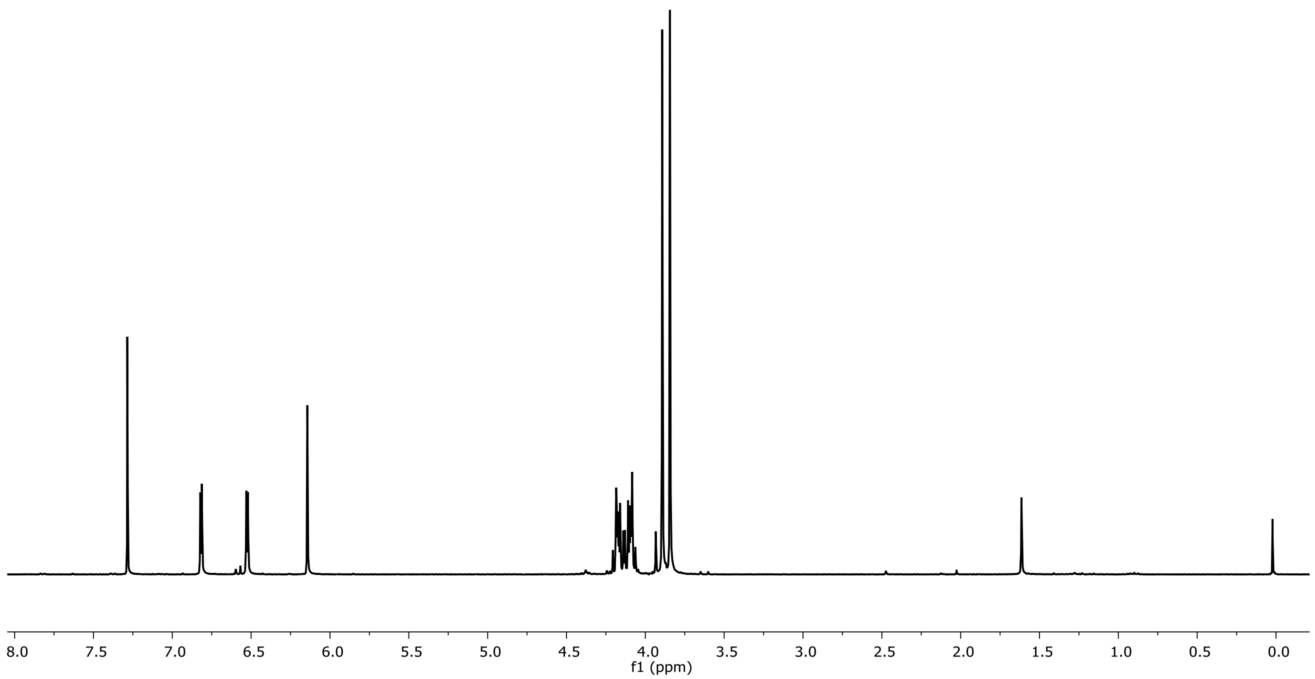

Supplementary Figure S4. ^{1}H NMR spectrum (300 MHz, $CDCl_3$) of **3**.

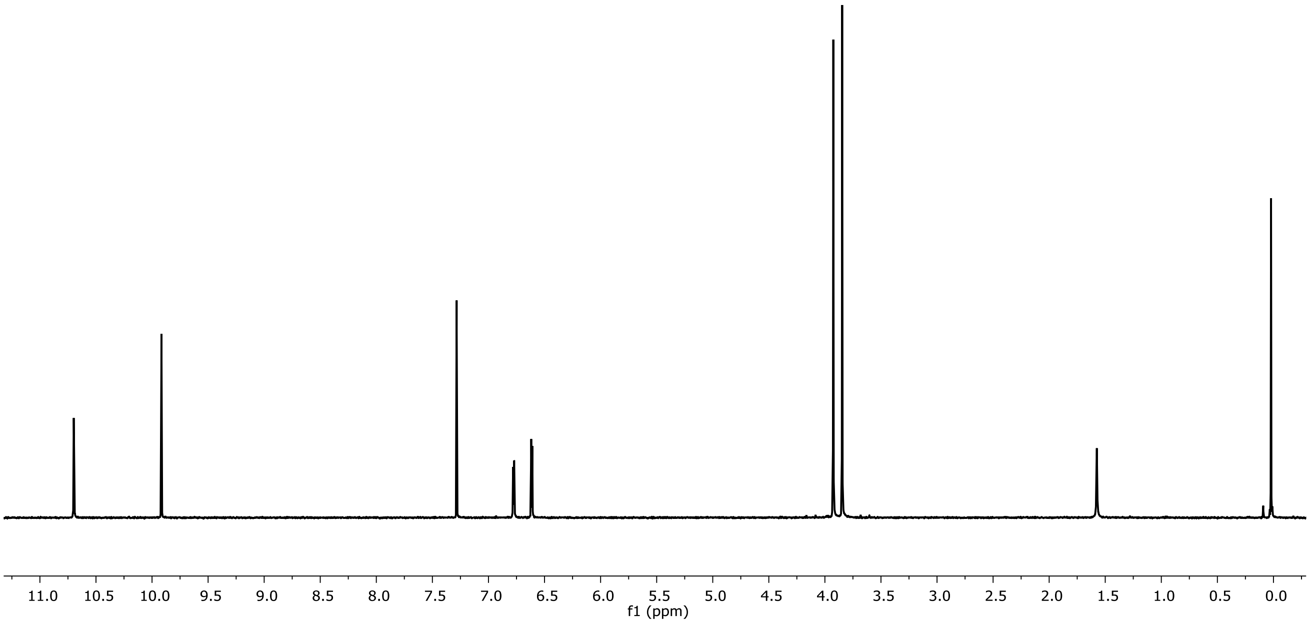

Supplementary Figure S5. ^{1}H NMR spectrum (300 MHz, $CDCl_3$) of **4**.

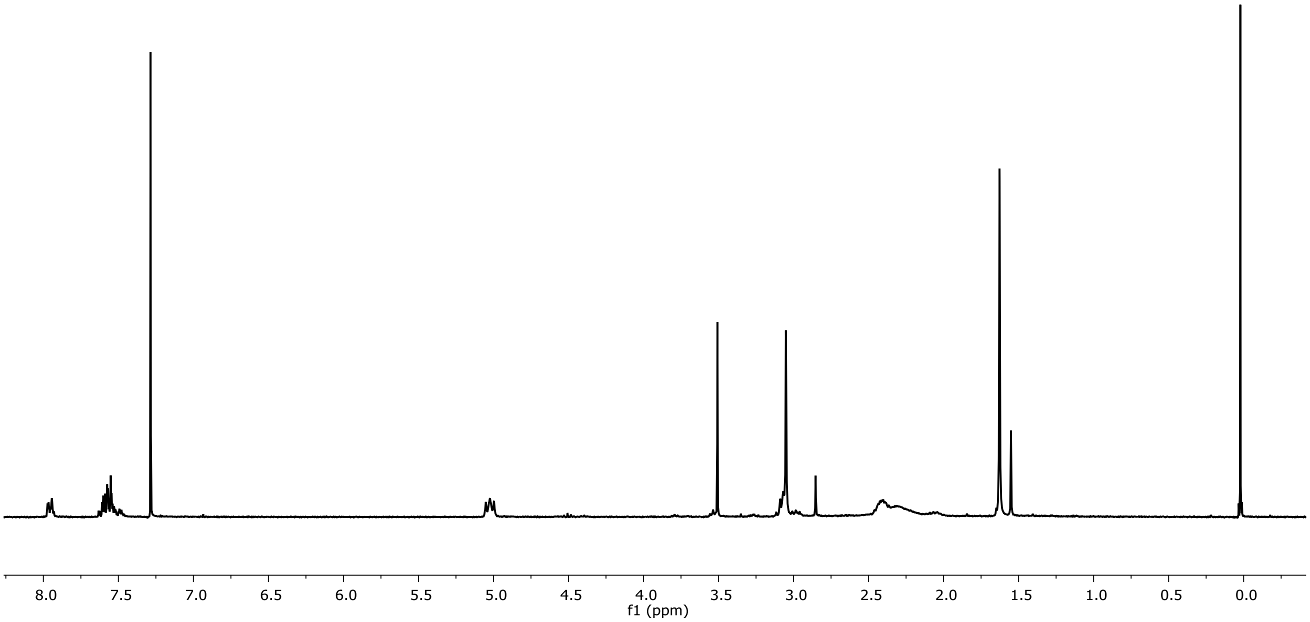

Supplementary Figure S6. ^{1}H NMR spectrum (360 MHz, $CDCl_3$) of **5**.

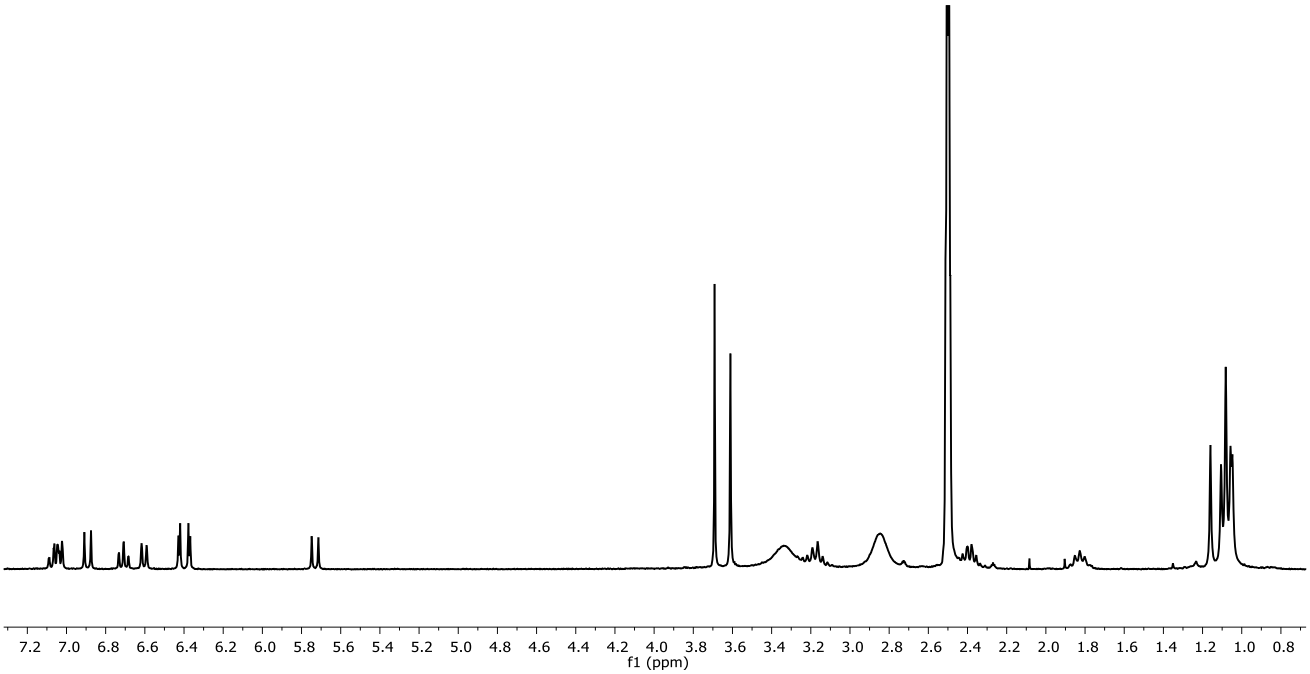

Supplementary Figure S7. ^{1}H NMR spectrum (300 MHz, DMSO-d_6) of **SP3**.

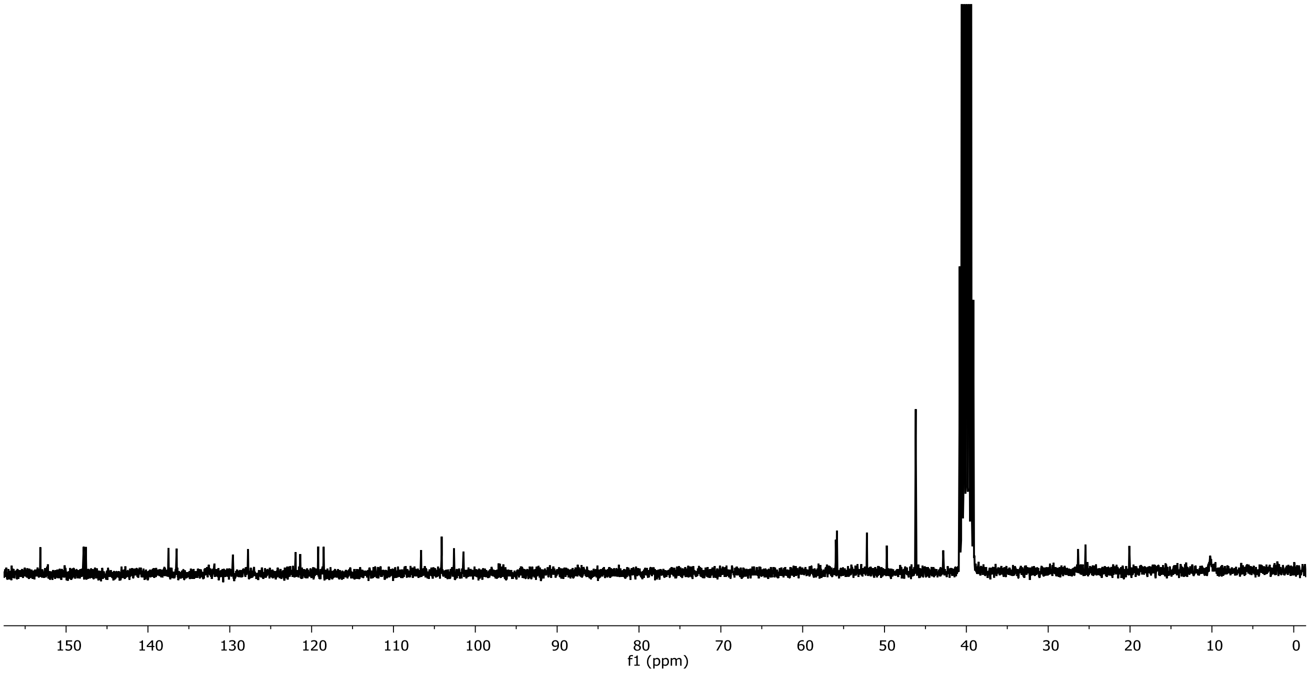

Supplementary Figure S8. ^{13}C NMR spectrum (75 MHz, DMSO-d_6) of **SP3**.

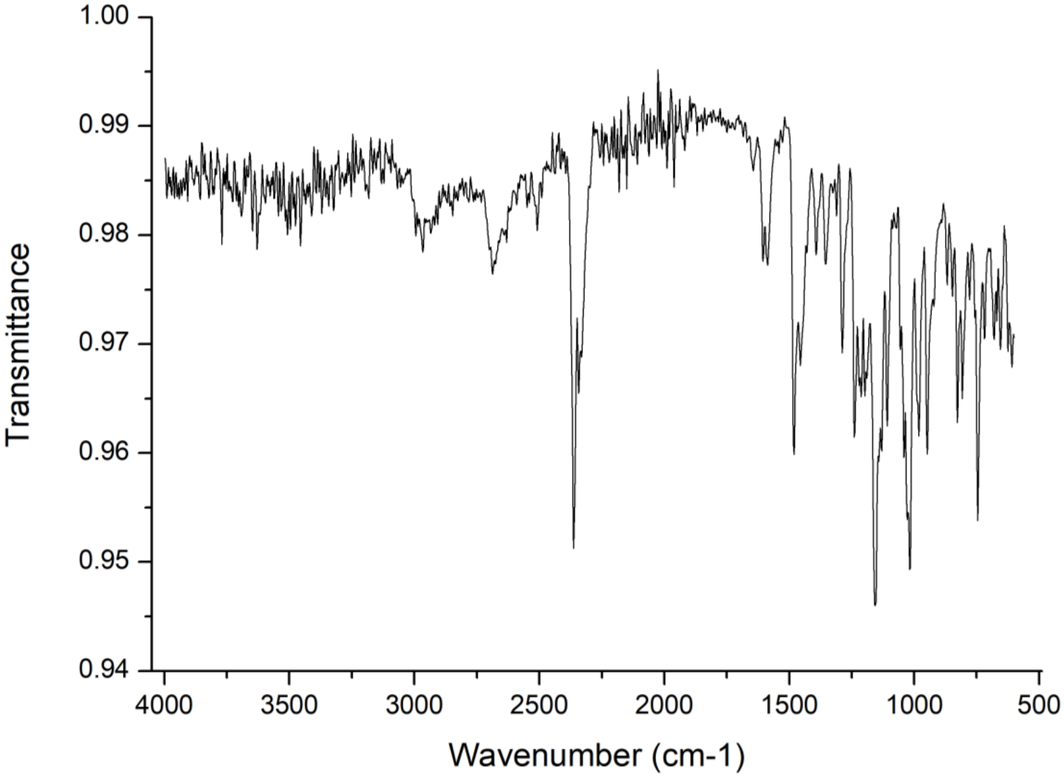

Supplementary Figure S9. IR (ATR) spectrum of **SP3**.

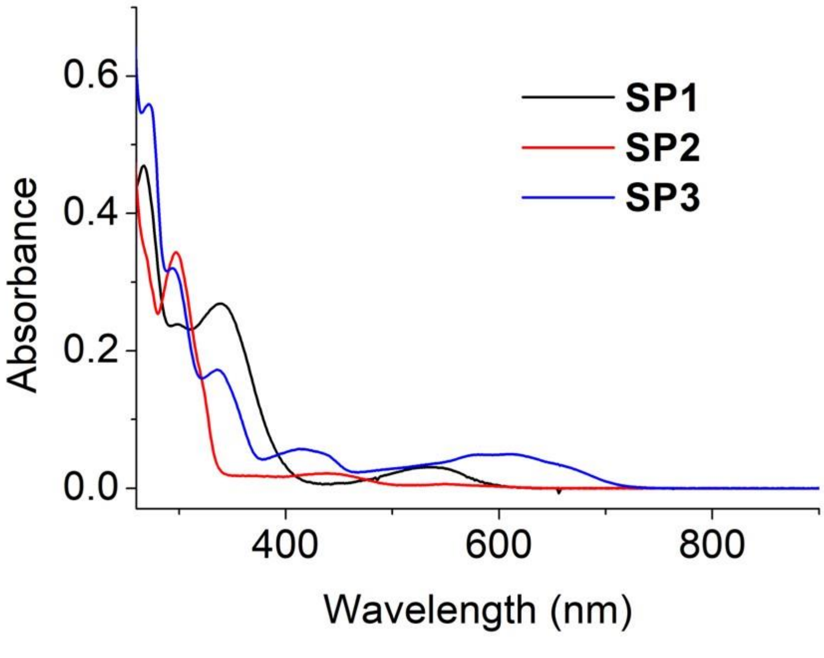

Supplementary Figure S10. UV-vis absorption spectrum of **SP1** (c = 1.0 10^{-5} M), **SP2** (c = 4.7 10^{-5} M) and **SP3** (c= 2.9 10^{-5} M) in MeOH.

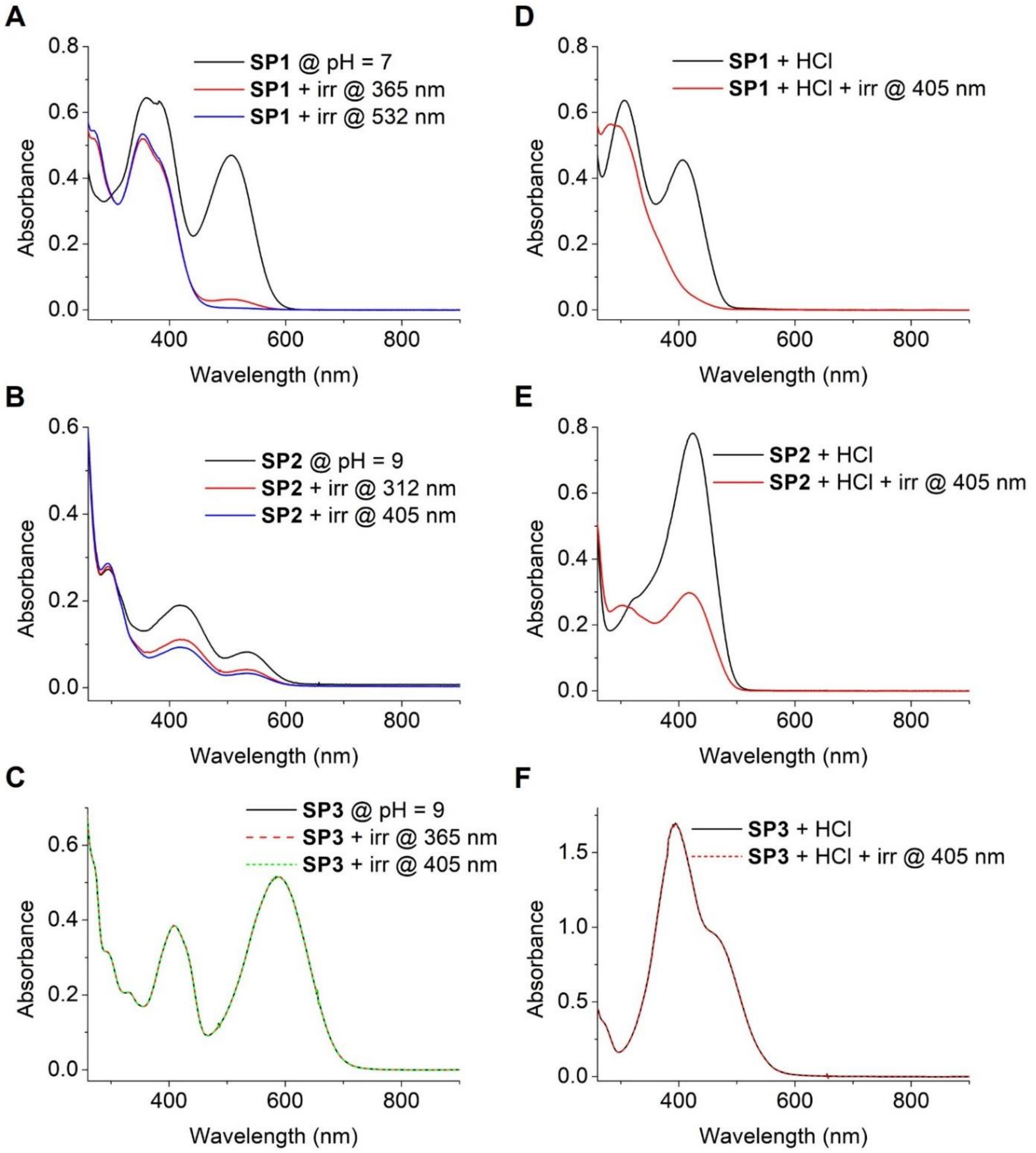

Supplementary Figure S11. (A-C) UV-vis absorption spectra of (**A**) **SP1** (c = 1.7 10^{-5} M), (**B**) **SP2** (c = 4.7 10^{-5} M) and (**C**) **SP3** (c = 3.6 10^{-5} M) in water before and after irradiation with UV or visible light. Measurements for **SP1** were conducted at pH = 7, as only the nonprotonated Sp and Mc isomers coexist at this condition. To accomplish the same situation for **SP2** and **SP3**, the aqueous media was slightly basified (pH = 9). (**D-F**) UV-vis absorption spectra of (**D) SP1** (c = 1.7 10^{-5} M), (**E**) **SP2** (c = 2.5 10^{-5} M) and (**F**) **SP3** (c = 3.6 10^{-5} M) in acid aqueous media before and after irradiation at 405 nm. In all the cases, sufficient HCl was initially added as to warrant full protonation of the spiropyrans to yield their McH form. The spectral changes observed for **SP1** and **SP2** upon irradiation are due to photoisomerization towards its *cis*-McH isomer, which absorbs at shorter wavelengths than McH and can further deprotonate to produce Sp because of its higher acidity (see **Scheme S1**) (Wimberger et al., 2021).

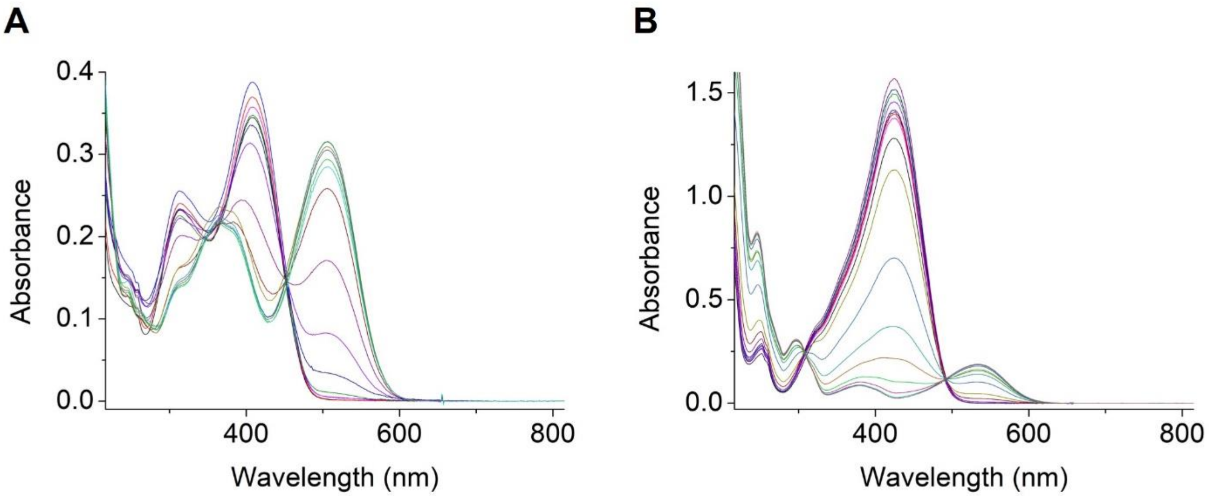

Supplementary Figure S12. Variation of the UV-vis absorption of (**A**) **SP1** and (**B**) **SP2** in water with pH. Measurements were conducted by dissolving **SP1** (c = 1.2 10^{-5} M) and **SP2** (c = 4.7 10^{-5} M) in independent buffer solutions of pH = 1.13, 1.59, 2.13, 2.64, 3.08, 3.61, 4.13, 4.72, 5.32, 5.78, 6.39, 6.84, 7.18, 7.56, 8.08, 8.69, 8.81, 9.57, 10.23 and 10.71.

Supplementary Table S1. Thermal equilibrium constants of spiropyrans **SP1**, **SP2** and **SP3**.[a]

	pK_a(McH)[b]	$pK_{a,intrinsic}$(McH)[b]	pK_a(*cis*-McH)[b]	K_c
**SP1**	4.63 ± 0.02	4.82 ± 0.21	1.56 ± 0.11	0.55[c]
**SP2**	6.23 ± 0.03	7.03 ± 0.82	2.28 ± 0.08	5.2[c]
**SP3**	6.17 ± 0.04	6.66 ± 0.52	- [d]	2.05[d]

[a] Constants are given for the different equilibria between Sp, Mc, McH and *cis*-McH (see **Supplementary Scheme S1**): (a) pK_a(McH), which is the experimental constant for the observed (Sp+Mc)-McH acid-base equilibrium; (b) $pK_{a,intrinsic}$(McH), which is the constant for the intrinsic Mc-McH acid-base equilibrium that was calculated as described in the literature (Berton et al., 2020; Wimberger et al., 2021); (c) pK_a(*cis*-McH), which is the experimental constant for the observed Sp-*cis*-McH equilibrium under irradiation at 405 nm; and (d) K_c, which is the experimental constant for the thermal Sp-Mc equilibrium in the dark. [b] Standard errors calculated from 4 replicates. [c] Values extracted from literature (Wimberger et al., 2021). [d] Not determined because we did not observe photoisomerization from McH to *cis*-McH. [e] Value calculated from the ^{1}H NMR spectrum of a solution of **SP3** in D_2O at pH = 9.00.

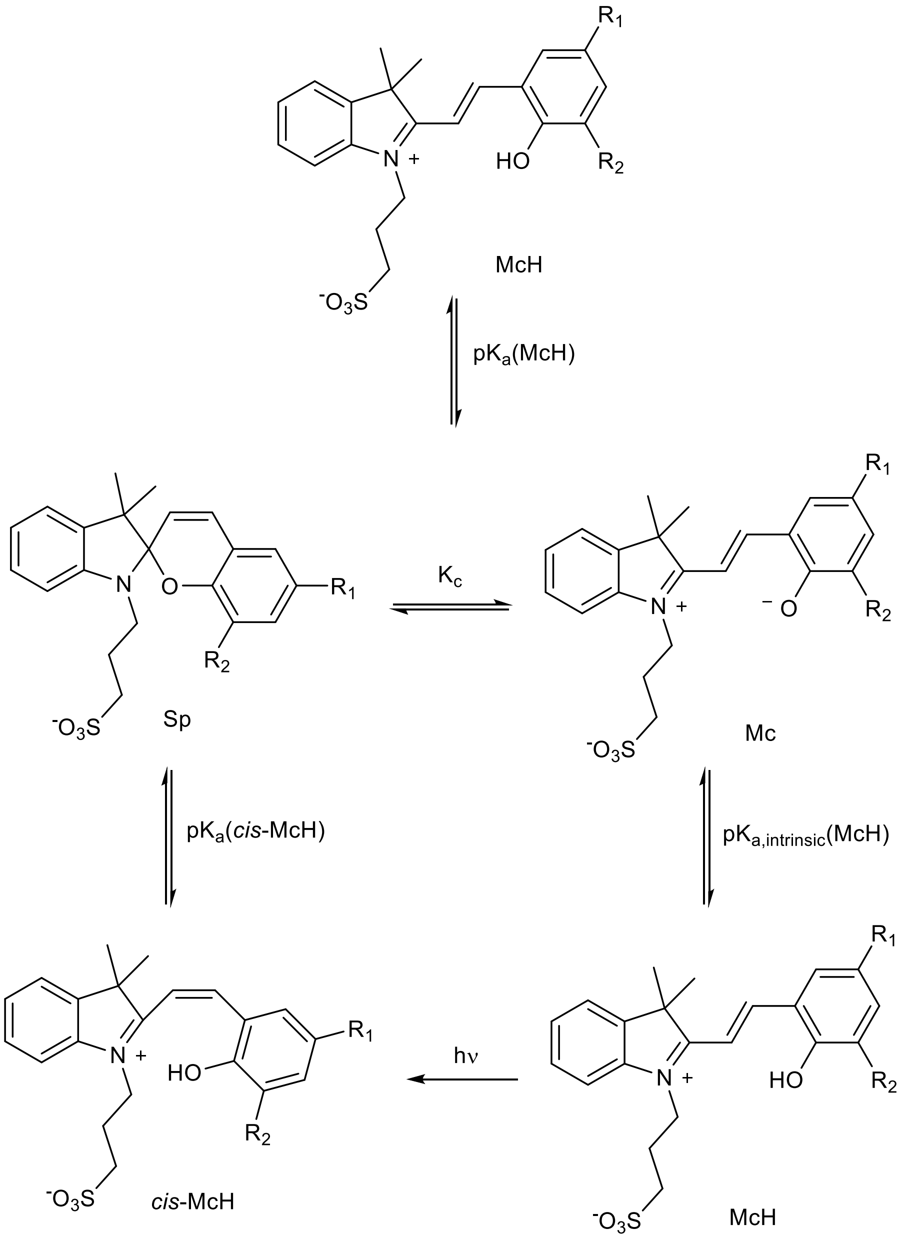

SP1 R_1 = NO_2, R_2 = H
SP2 R_1 = H, R_2 = H
SP3 R_1 = OMe, R_2 = OMe

Supplementary Scheme S1. Different equilibria of SPs that take place in water in the dark or after irradiation, which results in the formation of different states: Sp, Mc, McH and *cis*-McH. For **SP3**, we did not observe photoisomerization from McH to *cis*-McH.

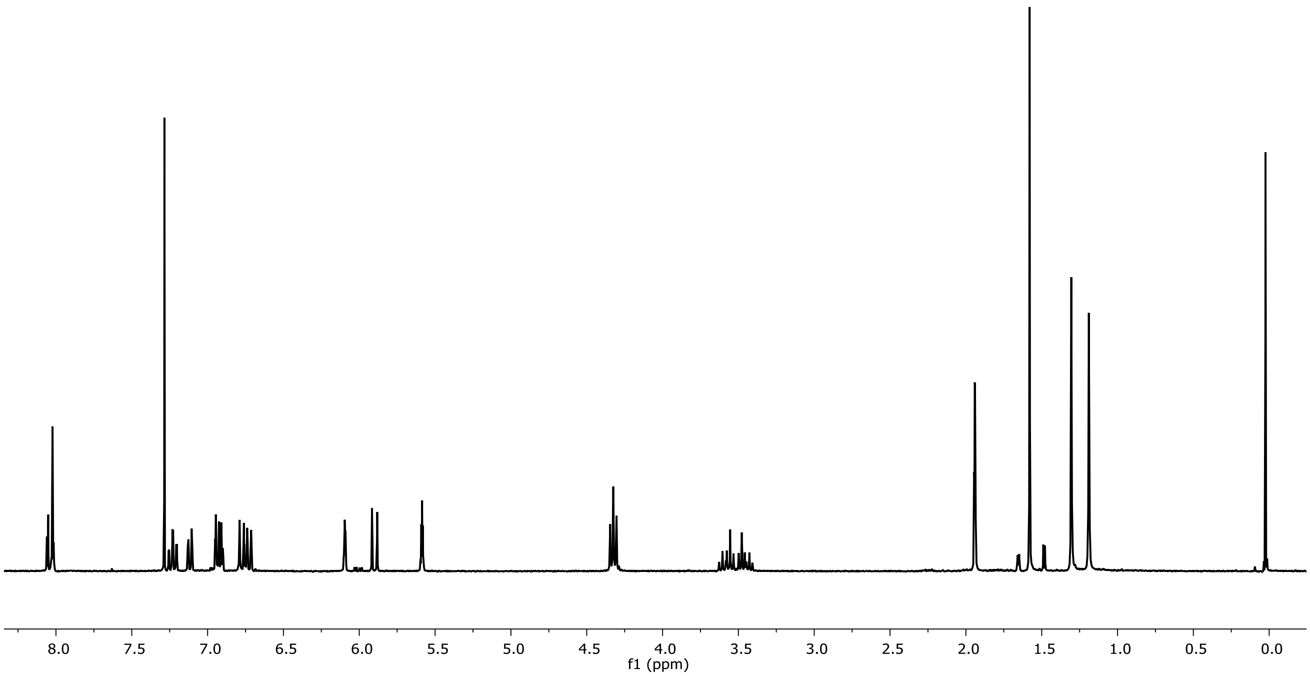

Supplementary Figure S13. ^{1}H NMR spectrum (300 MHz, $CDCl_3$) of **SP4**.

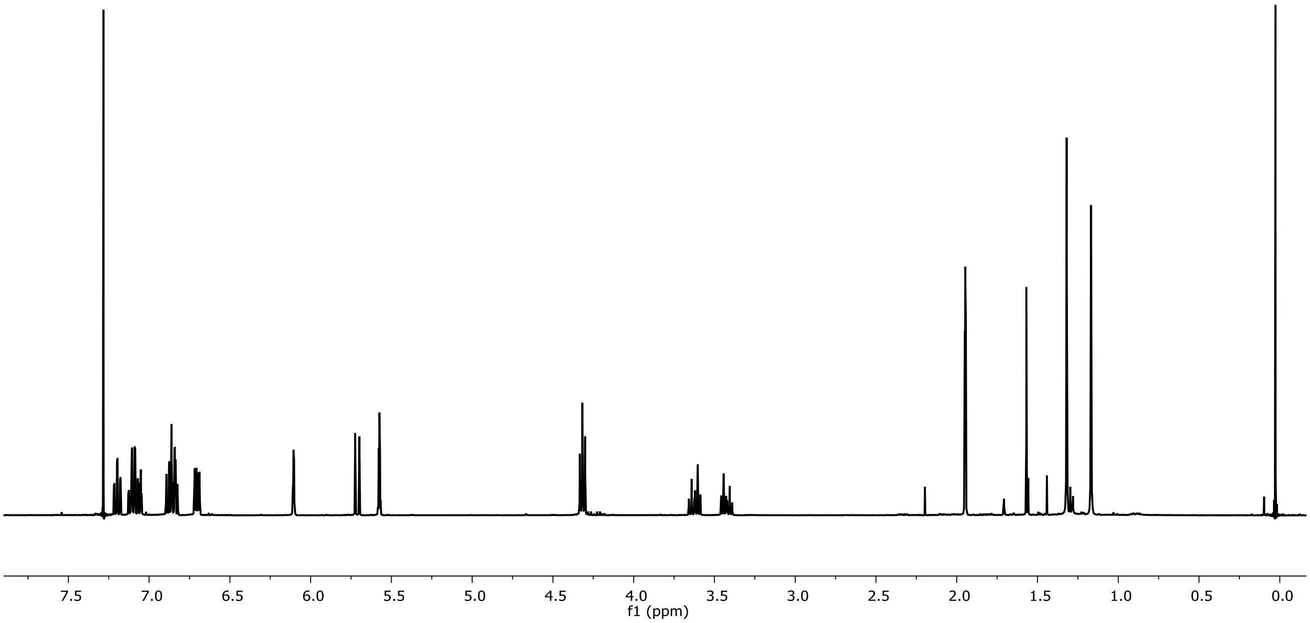

Supplementary Figure S14. ^{1}H NMR spectrum (300 MHz, $CDCl_3$) of **SP5**.

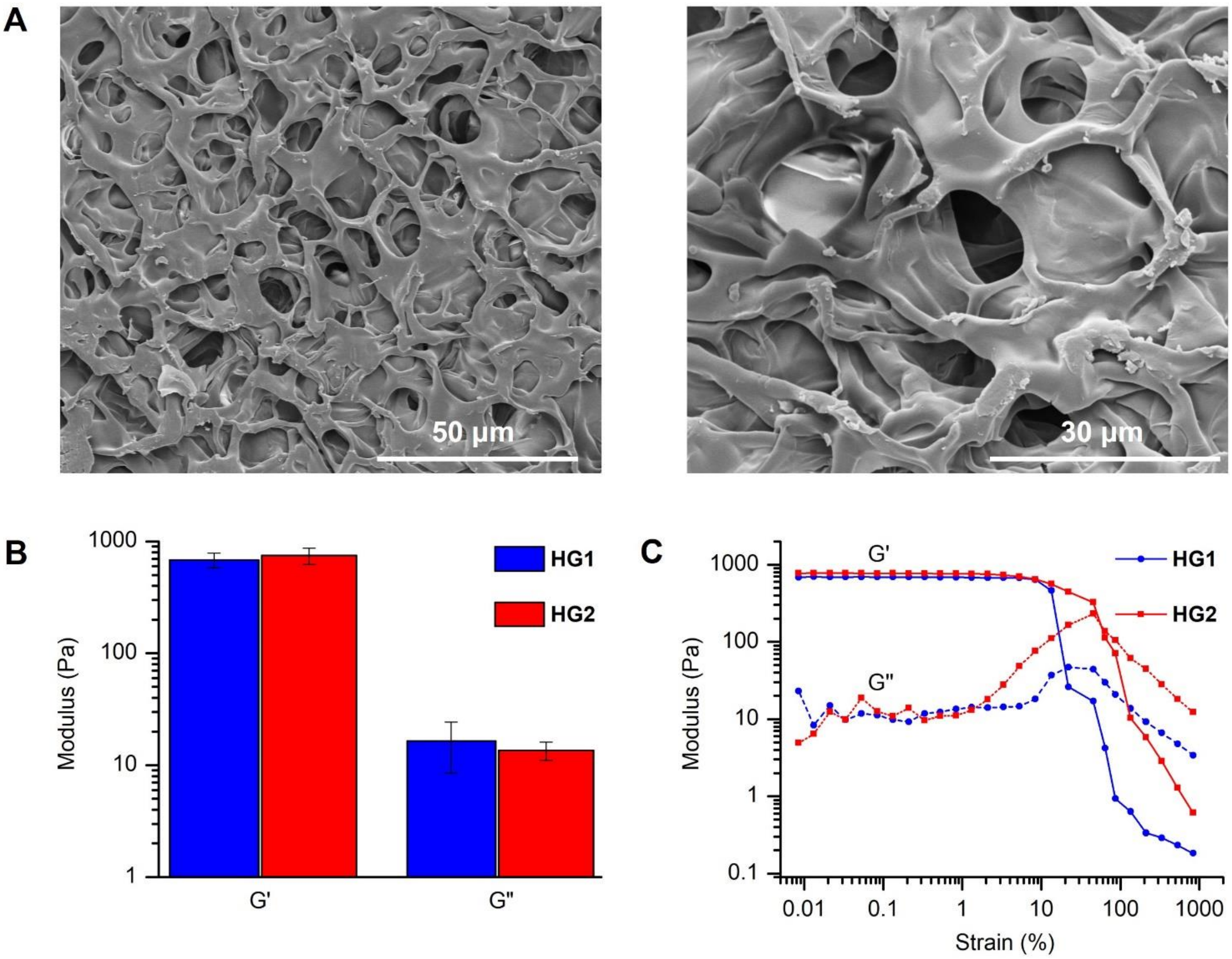

Supplementary Figure S15. (**A**) SEM images of **HG2** after freeze-drying, which reveal the hollow pore structure of our acrylamide gels. (**B**) Average values of the storage (G’) and loss (G”) moduli of **HG1** (G’ = 683 ± 102 Pa; G” = 16 ± 8 Pa) and **HG2** (G’ = 747 ± 125 Pa; G” = 14 ± 2 Pa). Errors bars are the standard deviation of the mean for 5 independent measurements on different hydrogels. (**C**) Strain sweep measurements of two representative samples of **HG1** and **HG2**.

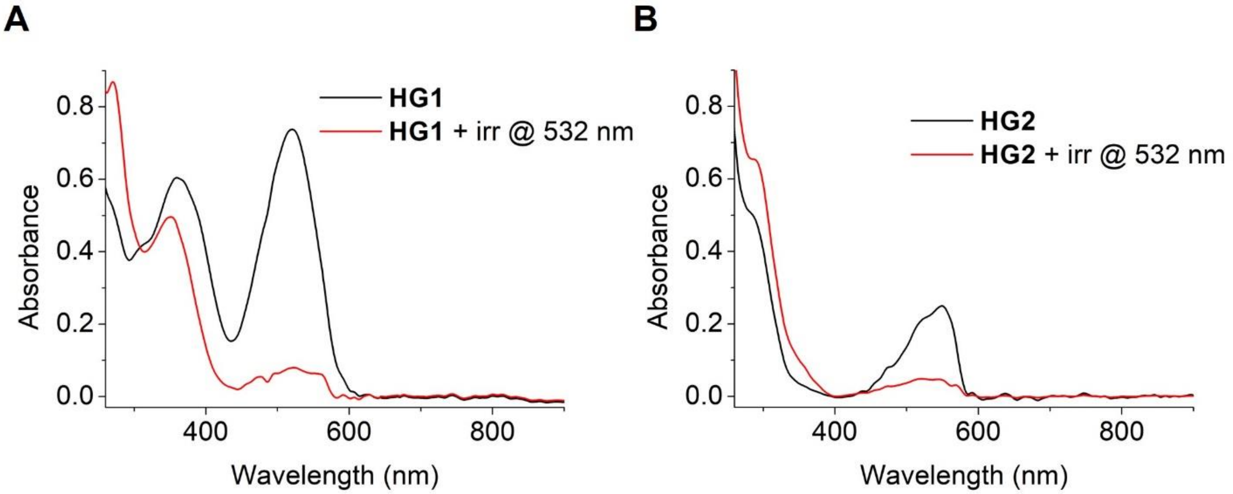

Supplementary Figure S16. Variation of the UV-vis absorption spectrum of (**A**) **HG1** and (**B**) **HG2** after irradiation at 532 nm.

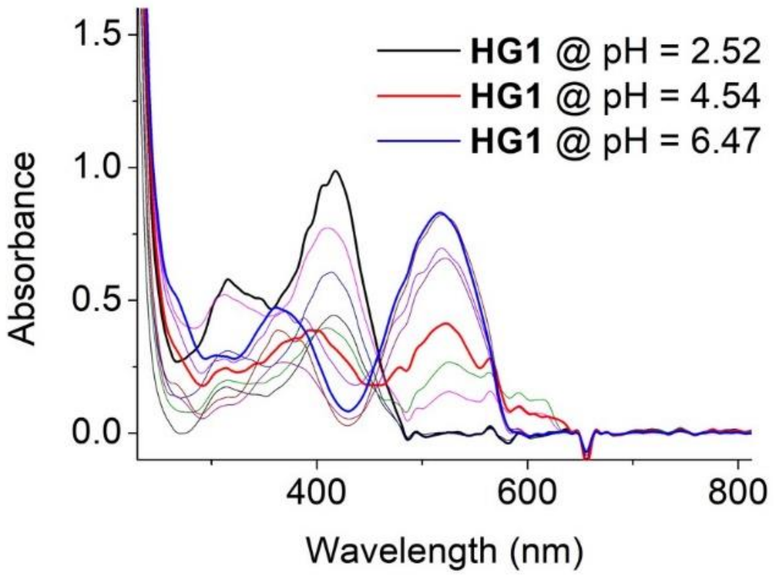

Supplementary Figure S17. Variation of the UV-vis absorption of **HG1**. Measurements were conducted by immersing **HG1** in independent buffer solutions of pH = 2.00, 2.52, 2.96, 3.48, 3.99, 4.54, 4.97, 5.49, 5.96 and 6.47.

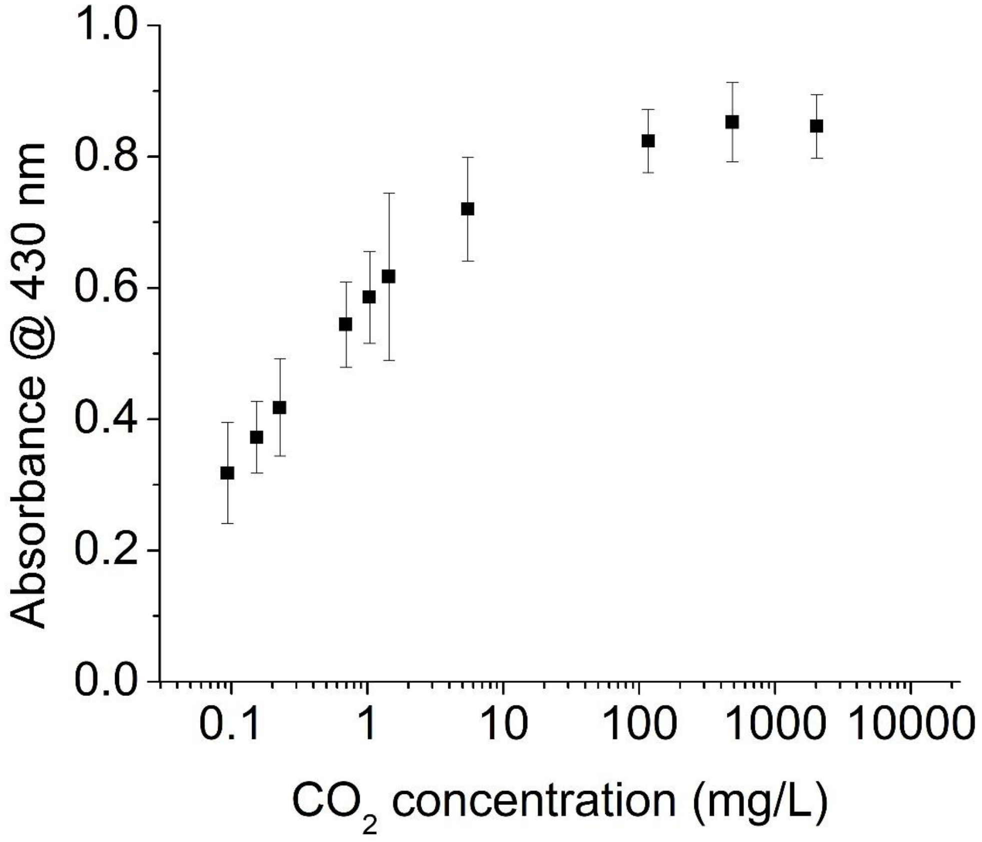

Supplementary Figure S18. Variation of the absorbance of **HG2** at λ = 430 nm for different concentrations of CO_2 absorbed by the gel when subjected to different times of exposure to a regular flux of gaseous CO_2 (5 mL min^{-1}). Error bars show the standard errors of the mean for 5 replicates measured in different hydrogels. To estimate the CO_2 concentration within the gel for each exposure time, we did the following: (a) we first assigned a pH value to each absorbance change based on the pH-dependent measurements previously registered for **HG2** (see Figure 5 in the main text); (b) we then determine the CO_2 concentration required to achieve each of these pH values based on the well-known acidity constants of CO_2 (pK_{a1} = 6.36) and HCO_3^- (pK_{a2} = 10.25) in pure water at 25 ºC and atmospheric pressure and the autoionization constant of water at these conditions (pK_w = 14).

2. REFERENCES

Berton, C. Busiello, D. M. Zamuner, S. Solari, E. Scopelliti, R., Fadaei-Tirani, F., Severin, K. and Pezzato, C. (2020). Thermodynamics and kinetics of protonated merocyanine photoacids in water. *Chem. Sci.* 11, 8457−8468. Available online at https://doi.org/10.1039/D0SC03152F.

Wimberger, L., Prasad, S. K. K., Peeks, M. D., Andréasson, J., Schmidt, T. W. and Beves, J. E. (2021). Large, tunable, and reversible pH changes by merocyanine photoacids. *J. Am. Chem. Soc.* 143, 20758-20768. Available online at https://doi.org/10.1021/jacs.1c08810.
